# Deep Multitask Learning-Driven Discovery of New Compounds Targeting *Leishmania infantum*

**DOI:** 10.1021/acsomega.4c07994

**Published:** 2024-12-16

**Authors:** Eder Soares de Almeida Santos, Jade Milhomem Lemos, Alexandra Maria dos Santos Carvalho, Felipe da Silva Mendonça de Melo, Eufrasia de Sousa Pereira, José Teófilo Moreira-Filho, Rodolpho de Campos Braga, Eugene N. Muratov, Philippe Grellier, Sébastien Charneau, Izabela Marques Dourado Bastos, Bruno Junior Neves

**Affiliations:** † Laboratory of Cheminformatics, Faculty of Pharmacy, Universidade Federal de Goiás, Goiânia 74605-170, Brazil; ‡ Pathogen-Host Interface Laboratory, Department of Cell Biology, Institute of Biological Sciences, Universidade de Brasilia, Brasilia 70910-900, Brazil; § InsilicAll Ltda, São Paulo 04571-010, Brazil; ∥ Laboratory for Molecular Modeling, UNC Eshelman School of Pharmacy, The University of North Carolina at Chapel Hill, Chapel Hill, North Carolina 27599-7360, United States; ⊥ UMR 7245 Molécules de Communication et Adaptation des Micro-organismes, Muséum National d’Histoire Naturelle, Équipe Parasites et Protistes Libres, Paris 75005, France; # Laboratory of Protein Chemistry and Biochemistry, Department of Cell Biology, Institute of Biological Sciences, Universidade de Brasilia, Brasilia 70910-900, Brazil

## Abstract

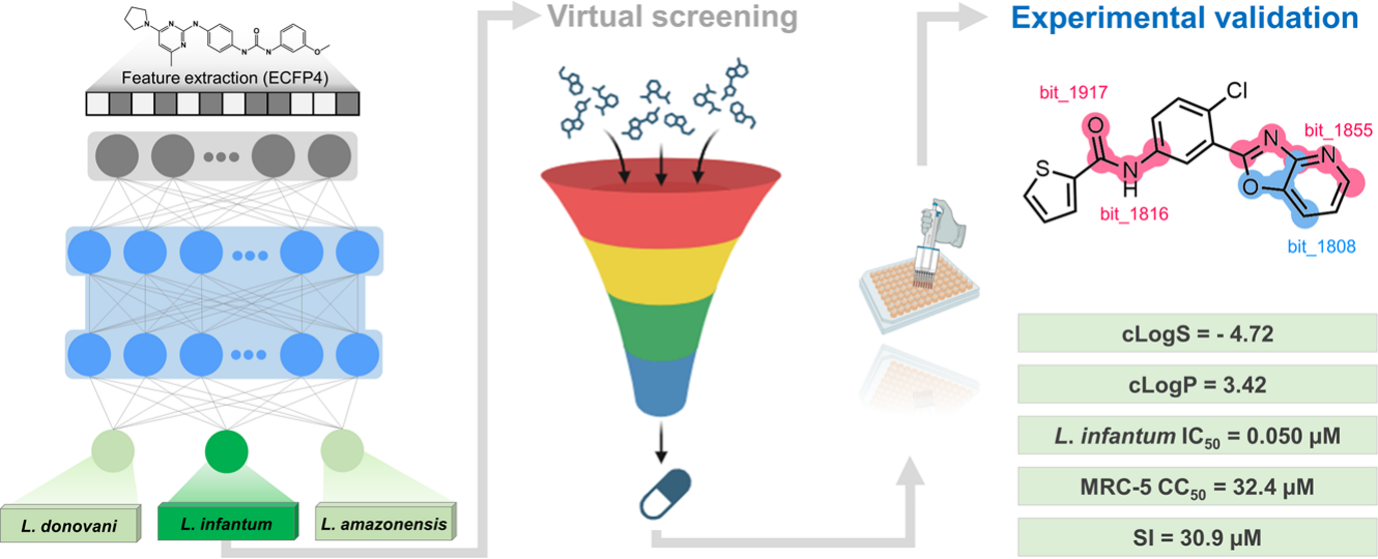

Visceral leishmaniasis caused by *Leishmania infantum* is a severe and often fatal disease prevalent in low- and middle-income countries. Existing treatments are hampered by toxicity, high costs, and the emergence of drug resistance, highlighting the urgent need for novel therapeutics. In this context, we developed an explainable multitask learning (MTL) pipeline to predict the antileishmanial activity of compounds against three *Leishmania* species, with a primary focus on *L. infantum*. Then, we screened ∼1.3 million compounds from the ChemBridge database by using these models. This approach identified 20 putative hits, with nine compounds demonstrating significant *in vitro* antileishmanial activity against *L. infantum*. Three compounds exhibited notable potencies (IC_50_ of 1.05–15.6 μM) and moderate cytotoxicities (CC_50_ of 32.4 to >175 μM), positioning them as promising candidates for further hit-to-lead optimization. Our study underscores the effectiveness of multitask learning models in virtual screening, enabling the discovery of potent and selective antileishmanial compounds targeting *L. infantum*. Incorporating explainable techniques offers critical insights into the structural determinants of biological activity, aiding in the rational design and optimization of new therapeutics. These findings advocate for the potential of multitask learning methodologies to enhance hit rates in drug discovery for neglected tropical diseases.

## 1 Introduction

Visceral leishmaniasis (VL) is a potentially fatal vector-borne disease mainly caused by the two protozoa of the genus *Leishmania*: *L. donovani* (Africa and Asia) and *L. infantum* (Latin America and the Middle East).^1−3^ It is characterized by irregular bouts of fever, weight loss, enlargement of the spleen and liver, and anemia. Over 200 million people residing in 70 countries are at risk of VL infection. However, Brazil, East Africa, and India account for 90% of the new human cases. An estimated 500,000 new cases of VL and 50,000 deaths occur annually, which are thought to be underestimated.^4^ Without an effective vaccine for humans, disease treatment mainly relies on effective chemotherapy. However, currently available treatments are inadequate because of high costs, long-term and complex protocols, toxicity, drug resistance, and the need for parenteral administration.^5,6^ Therefore, novel drug discovery approaches are needed to find practical, innovative drugs for this disease.

In recent decades, data science has significantly transformed drug discovery, allowing for the rapid *in silico* exploration of vast chemical spaces and enhancing the probability of identifying novel chemotypes with desirable properties.^7−9^ Notably, the prioritized compounds predicted by virtual screening often exhibit advantageous hit rates of 10–40% in experimental testing, yielding novel hits with potencies in the nanomolar to low micromolar range.^10,11^ This hit rate is considerably higher than those typically achieved through traditional high-throughput screening and DNA/RNA-tagged library screening methods. These usually yield hit rates below 0.1%, emphasizing the efficiency of computational approaches in drug discovery.^10,11^

Through recent advancements in hardware accelerators, drug discovery pipelines have been significantly refined by using deep neural networks (DNNs) with flexible architectures.^12^ These networks are up-and-coming for predicting antiparasitic activity against challenging pathogens like *L. infantum*. DNNs excel in learning high-level chemical features from extensive data sets, which often comprise thousands to millions of compounds.^12−14^ By leveraging deep learning techniques, these networks can identify intricate patterns and relationships within the chemical data that traditional methods might overlook. The representations generated by DNNs effectively map chemical structures into continuous vectors in a high-dimensional space. These vectors encapsulate critical information about the chemical and physical properties of the compounds. Subsequently, these detailed and nuanced representations are used to predict the biological properties of new, untested compounds.^15−17^

Although DNNs have enormous potential to establish complex quantitative structure–activity relationships (SAR), the predictive performance of these methods is typically dependent on a large amount of data. Furthermore, DNNs are sometimes called “black box” models because their decision-making mechanisms are not explicitly accessible to human cognition.^18^ To alleviate these problems, our research group has been developing explainable multitask learning (MTL) models that can enhance the predictive performance of tasks with limited data and interpretability to the predictions.^19^ MTL aims to simultaneously learn multiple related tasks, allowing the knowledge acquired from one task to benefit others, thereby enhancing the generalization performance across all tasks. By integrating data from various tasks, MTL effectively increases the overall data set size, enabling the model to learn more robust and universal representations. This larger data set leads to improved knowledge sharing among tasks, superior performance in each task, and a reduced risk of overfitting.^20−22^

This study developed multitask classification and regression models to predict compounds’ antileishmanial activity across three *Leishmania* species, explicitly focusing on enhancing predictive power for the target *L. infantum*, which is the primary causative agent of visceral leishmaniasis in Latin America. These models leveraged shared knowledge in scarce and large-data scenarios, resulting in improved performance compared with single-task models. Subsequently, the best multitask models were employed to screen 1.3 million compounds from the ChemBridge database. The most promising compounds were tested *in vitro* against *L. infantum*, leading to the discovery of novel early stage *L. infantum* hits.

## 2 Materials and Methods

### 2.1 Computational

#### 2.1.1 Data Collection and Curation

Compounds with half maximal inhibitory concentration (IC_50_) data (72 h exposure) against amastigotes of *L. donovani* (CHEMBL367), *L. infantum* (CHEMBL612848), and *L. amazonensis* (CHEMBL612877) were retrieved from the ChEMBL database.^23−26^ To ensure numerical stability in regression model development, IC_50_ values (μM) were converted to their negative logarithmic scale, pIC_50_. Classification models were established using an activity threshold of 10 μM to differentiate between active (pIC_50_ ≥ 5) and inactive (pIC_50_ < 5) compounds.^27^ Chemical structures in simplified molecular-input line-entry system (SMILES) format and corresponding bioactivity data were meticulously curated following the guidelines of Fourches et al.,^28−30^ including the normalization of nitro groups and aromatic rings and exclusion of salts, mixtures, polymers, and organometallic compounds. Finally, the data set was thoroughly analyzed, and duplicates were identified through InChIKey and removed as follows:

- Classification: (i) if duplicates presented discordance in bioactivity outcomes (e.g., active vs inactive), both entries would be excluded; and (ii) if the reported outcomes of the duplicates were the same, one entry would be retained in the data set and the other excluded.
- Regression: (i) duplicates were inspected visually; (ii) if duplicates presented discordant bioactivities, i.e., pIC_50_ values >0.3 log units, both entries would be excluded; and (iii) if the reported pIC_50_s were ≤0.3 log units, an average of the pIC_50_ values was calculated, and one entry was retained in the data set.

#### 2.1.2 Similarity Maps

Curated data sets were analyzed by generating similarity maps using DataWarrior v.05.02.01.^31^ This map employs the Rubberbanding Force field technique to depict the similarity (vertices) among compounds (nodes). The process involves several stages: (i) initial random placement of all compounds in a two-dimensional space; (ii) calculation of a similarity matrix for all compounds using Tanimoto coefficients (Tc) and FragFP descriptors; (iii) identification of the closest neighbors (Tc > 0.8) for each compound; and (iv) iterative adjustment of the compounds’ positions to ensure that molecules with higher similarity are positioned closer to one another.^31^

#### 2.1.3 Structure Representation

Before model building, the standardized SMILES strings were encoded into either extended connectivity fingerprints with a diameter of 4 (ECFP4)^32^ or molecular graphs using the RDKit package v.2020.03.1^33^ in Python v.3.8.^34^ The ECFP4 fingerprints were generated as bit vectors containing 2048 bits. Molecular graphs were created by incorporating a range of atom features (e.g., element symbol, valence electron count, hydrogen bond count, and orbital hybridization) and bond features (e.g., bond type, conjugation).

#### 2.1.4 Split of Data Sets

The curated data sets were imported into Python and randomly divided into training, validation, and test sets in an 8:1:1 ratio. The training set was used to build the model, the validation set was used for hyperparameter optimization, and the test set was used for model evaluation. In addition to random splitting, a scaffold splitting method available in Chemprop^35,36^ was utilized to ensure that the training, validation, and test sets did not share the same Bemis-Murcko scaffolds.^37^

#### 2.1.5 Multitask Learning Protocol

Multitask learning models based on deep neural network (MT-DNN) and message-passing neural network (MT-MPNN) architectures were developed to simultaneously predict the antileishmanial activity of untested compounds against three species, with a primary focus on improving generalization for *L. infantum*. The MT-DNN model used ECFP4 fingerprints as input, while the MT-MPNN utilized feature vectors for the atoms and bonds within the molecules. Both models were implemented using Cuda v.10.1 on the GPU version of TensorFlow v.2.9. Due to the complexity and high computational cost of the architectures explored, the hyperparameters of models were optimized using a random search strategy based on previous experience. An early stopping approach was employed to prevent overfitting and conserve computational resources, with a maximum of 2000 epochs. The training was stopped early if the performance metric did not improve within 10 epochs on the validation set.

#### 2.1.6 Single-Task Learning Protocol

To enhance the evaluation of methodologies, the multitask models were benchmarked against single-task DNNs and MPNNs alongside three state-of-the-art Random Forest (RF) and LightGBM methods. RF and algorithm-based models were implemented using Scikit-learn v.0.24.2, while LightGBM models were developed using the LightGBM package. The RF and LightGBM models employed ECFP4 fingerprints as input features. These models were built using a 5-fold cross-validation approach, with hyperparameters optimized through a Bayesian method implemented in Scikit-Optimize v.0.7.4. To ensure consistent model performance comparisons, the integrity of all data sets (training and test sets) was maintained across the multitask models.

#### 2.1.7 Model Evaluation

The predictive performance of classification models was evaluated using accuracy (ACC), recall, specificity (SP), and the Matthews correlation coefficient (MCC). These metrics were calculated as follows
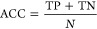
1
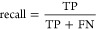
2
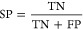
3
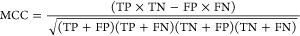
4where, *N* represents the number of compounds, FP and FN represent the number of false positives and false negatives, and TP and TN represent the number of true positives and true negatives, respectively. The predictive performance of regression models was evaluated using Pearson correlation coefficient (*r*), root-mean-square errors (RMSE), and mean absolute error (MAE), as follows
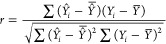
5
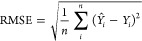
6
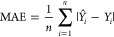
7where, *n* is the number of compounds; *Y*_*i*_ is the observed value for each particular compound; *Y̅*_*i*_ is the mean of observed values;  is the mean observed values over all compounds; and *Y̅* is the mean predicted values over all compounds. The statistical performance of the classification and regression models was reported as the mean results from five separate splitting runs.

#### 2.1.8 Model Explainability

Feature contributions for the antileishmanial activity were assessed following the SHapley Additive exPlanations (SHAP) values recently reported by Rodríguez-Pérez and Bajorath.^38^ SHAP values are a model-agnostic method for interpreting the predictions of any machine learning model. This algorithm was adapted for the MT-DNN regression model using the model-independent kernel SHAP approach. To interpret predictions for individual tasks, kernel SHAP calculations were carried out for each output unit (task) of the MT-DNN regression model. Then, multiple SHAP visualizations were combined to investigate the predictions against *L. infantum*.

### 2.2 Experimental Section

#### 2.2.1 Materials

Compounds were acquired from ChemBridge (San Diego, CA), dissolved in 100% dimethyl sulfoxide (DMSO) to achieve a stock concentration of 20 mM, and stored at −20 °C. The chemical structures of all compounds were verified using proton nuclear magnetic resonance (^1^H NMR) spectroscopy or liquid chromatography–mass spectrometry (LC-MS) analysis, which included evaporative light scattering and ultraviolet detectors. This analysis confirmed that all compounds had a minimum purity of 95% (spectra of the compounds are provided in the Supporting Information).

#### 2.2.2 Parasite Culture Conditions

*L. infantum* (MHOM/BR/2002/LPC-RPV) promastigotes were maintained in Schneider’s medium supplemented with 10% (v/v) fetal bovine serum (FBS, Gibco) and 100 U/mL penicillin and 100 μg/mL streptomycin at 28 °C. MRC-5 human primary fetal lung fibroblast cells were maintained in RPMI 1640 medium supplemented with 10% heat-inactivated fetal calf serum (FCS) and 25 mM HEPES under a 5% CO_2_ atmosphere at 37 °C and pH 7.4.

#### 2.2.3 *In Vitro* Antileishmanial Activity against Promastigotes

To evaluate the effect of prioritized compounds on parasite viability, 4 × 10^6^ promastigotes/mL were incubated in the presence of a series of 11 points from 200–0.195 or 50–0.04 μM plus control (0.1% DMSO) for 48 h at 28 °C to a final volume of 200 μL in 96-well microplate. Next, 20 μL of resazurin solution (0.39 mM) was added to each well and incubated for 4 h, and then fluorescence was recorded (570 nm_ex_/595 nm_em_) on a microplate reader SpectraMax M5 (Molecular Devices, Sunnyvale, CA). The IC_50_ from three independent experiments was calculated from the dose–response curve fitted in a four-parameter logistic, nonlinear regression model using GraphPad Prism software (CA) v.8.

#### 2.2.4 *In Vitro* Cytotoxicity Test

MRC-5 cells were seeded in 96-well plates at a density of 1 × 10^4^ cells/well using 100 μL of medium. After 24 h, compounds were serially diluted in another set of 96-well plates starting from a concentration of 250 μM (11 dilution points plus the control), using 100 μL of complete culture medium per well. Subsequently, 100 μL of these diluted compounds were transferred to the seeded cells. Amphotericin B (Sigma-Aldrich) dissolved in DMSO was used as a control and diluted from an initial concentration of 0.5 μM. After 72 h of incubation, resazurin was added to a final concentration of 45 μM and incubated for 4 h before measuring fluorescence. The cytotoxic concentrations causing 50% cell growth inhibition (CC_50_) were derived from data obtained from two independent experiments and calculated in the same manner as for IC_50_, as described above.

## 3 Results and Discussion

### 3.1 Bioactivity Data Analysis

We employed a deep multitask learning approach to advance the discovery of novel antileishmanial compounds targeting *L. infantum*. To build a robust model, we initially retrieved compounds with reported *in vitro* antileishmanial activity against amastigotes of *L. infantum*, *L. donovani*, and *L. amazonensis* from the ChEMBL database.^23−25^ Including multiple *Leishmania* species was essential to increase the overall data set size and leverage shared biological information across species, thereby enhancing the model’s predictive performance for the *L. infantum* task.

**Figure 1:**
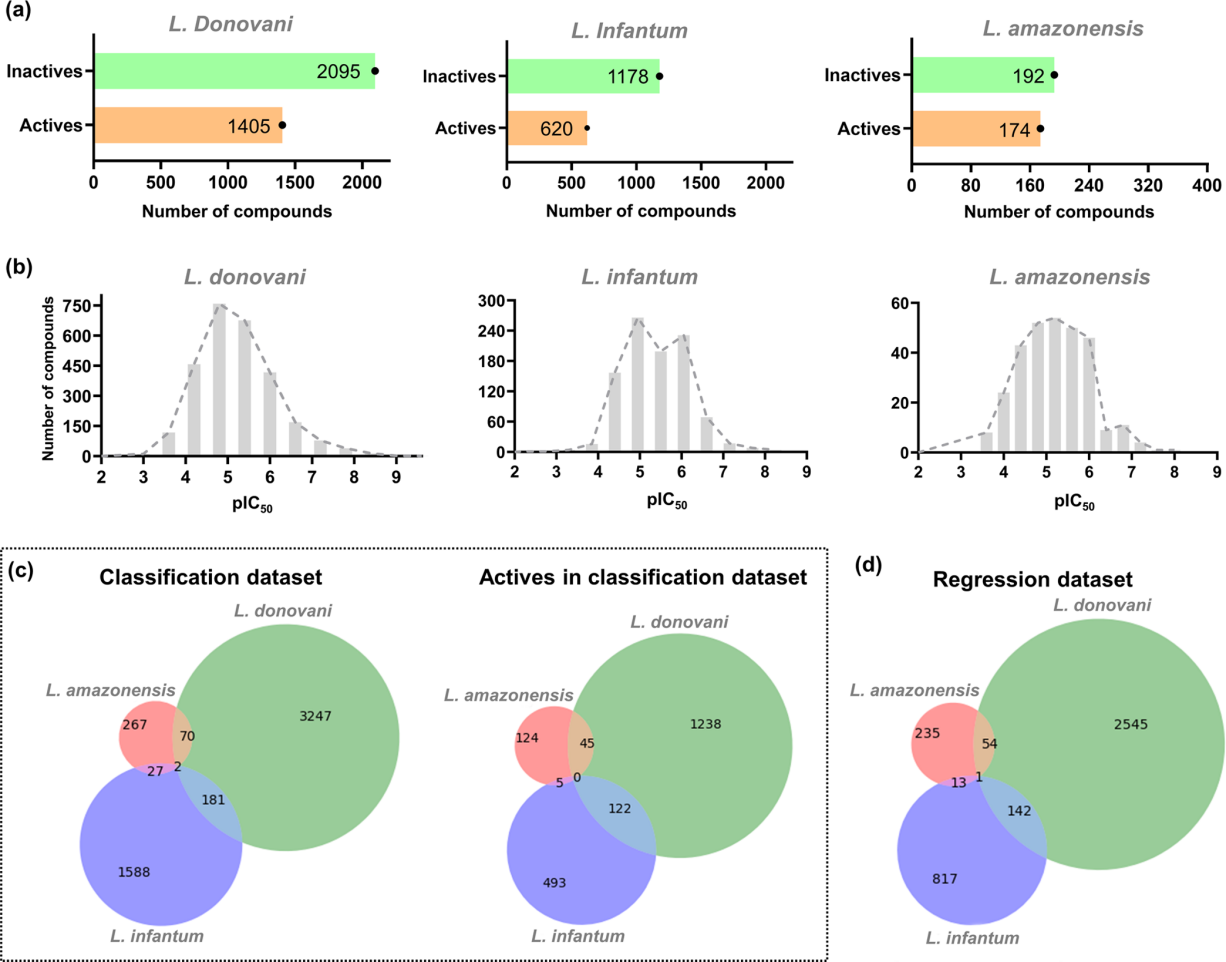
Bioactivity distribution of antileishmanial data in classification and regression data sets. (a) Bar graphs illustrating the number of actives and inactives in *L. donovani*, *L. infantum*, and *L. amazonensis* tasks. (b) Histograms illustrate the distribution of pIC_50_ values for each task. Venn diagram showing the content overlap between tasks in the (c) classification and (d) regression data sets.

Subsequently, an activity threshold of 10 μM was defined to discriminate between active and inactive compounds, representing a robust starting point for hit-to-lead optimization. This threshold ensures that selected compounds have sufficient potency to warrant further development, aligning with established criteria for advancing drug candidates in neglected disease research.^27^Figure 1a illustrates the number of active and inactive compounds obtained for each *Leishmania* species. The *L. donovani* task comprises 3500 compounds, with 1405 classified as actives and 2095 as inactives. In contrast, the *L. infantum* task includes 1798 compounds (620 actives and 1178 inactives), while the *L. amazonensis* task consists of 366 compounds (174 actives and 192 inactives).

Considering that a subset of compounds had complete IC_50_ curves, a total of 2742 compounds with reproducible potencies were retained for the *L. donovani* task, 973 compounds for the *L. infantum* task, and 303 compounds for the *L. amazonensis* task, to develop regression models (Figure 1b). These data sets encompassed a wide range of potencies, with pIC_50_ values ranging from 5623 μM (pIC_50_ = 2.25) to 0.00028 μM (pIC_50_ = 9.54), spanning 5–7 logarithmic units.

The activity outcomes and pIC_50_ values obtained from each species were combined to create multitask matrices. This resulted in a multitask classification data set containing 5382 compounds and a multitask regression data set with 3807 compounds. Merging these data sets increased the overall size and provided a more comprehensive collection of compounds, featuring diverse chemical structures and activity profiles for model development. Figure 1c illustrates the overlap of activity outcomes (left) and actives (right) across the *L. infantum*, *L. donovani*, and *L. amazonensis* tasks in the classification data set. Notably, *L. infantum* and *L. donovani* share 181 compounds, with 122 categorized as actives, underscoring the value of leveraging data from multiple *Leishmania* species to enrich the model’s training set. Furthermore, a similar overlap (142 compounds) was observed between *L. donovani* and *L. infantum* tasks in regression data sets (Figure 1d). In contrast, there is minimal overlap of compounds tested across all three species, reflecting the limited availability of compounds tested consistently across multiple *Leishmania* species.

Multitask learning is typically applied when there is a meaningful correlation between tasks. Given this, we calculated the concordance of activity outcomes and the Pearson correlation coefficients of pIC_50_ values between each pair of tasks. Supporting Figure 1a shows strong concordance (>0.74) between the activity outcomes for the *L. donovani*, *L. infantum*, and *L. amazonensis* tasks, confirming the consistent effectiveness of the 10 μM activity threshold used in this study. Furthermore, significant correlations ranging from 0.67 to 0.88 were observed among the pIC_50_ values across the three tasks.

### 3.2 Chemical Space Analysis

A chemical space analysis was conducted by using similarity maps to evaluate the structural diversity of the collected compounds and assess the potential for sharing chemical information across tasks. These similarity maps provide insights into each data set’s structural diversity and activity distribution. In the maps, nodes represent individual compounds, and edges depict similarity relationships computed using FragFP descriptors.^31^

**Figure 2:**
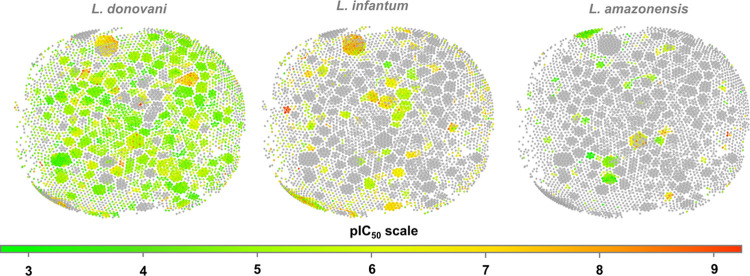
Similarity maps illustrating structural diversity and pIC_50_ values across the *L. donovani*, *L. infantum*, and *L. amazonensis* tasks. Nodes represent individual compounds, and edges indicate similarity based on FragFP descriptors. Gray nodes correspond to untested compounds.

As shown in Figure 2, the *L. donovani* task displays extensive coverage of the chemical space with clusters distributed across nearly all regions. This indicates a high degree of structural diversity and a wide range of bioactivity within the data set. In contrast, the *L. infantum* and *L. amazonensis* tasks show more localized clusters, reflecting a narrower chemical diversity and a more limited range of antileishmanial activity. These findings underscore the comprehensive nature of the *L. donovani* data set, which encompasses diverse chemical structures and activities, making it advantageous for antileishmanial drug discovery. Conversely, the constrained chemical space coverage in the *L. infantum* and *L. amazonensis* data sets suggests the need to expand their chemical diversity to improve the reliability of predictive models for these tasks. This underscores the advantage of a multitask learning approach, as including *L. donovani* data enriches the chemical space and enhances the overall predictive power, benefiting all tasks by leveraging shared chemical information across species.

### 3.3 Multitask Learning Models

**Figure 3:**
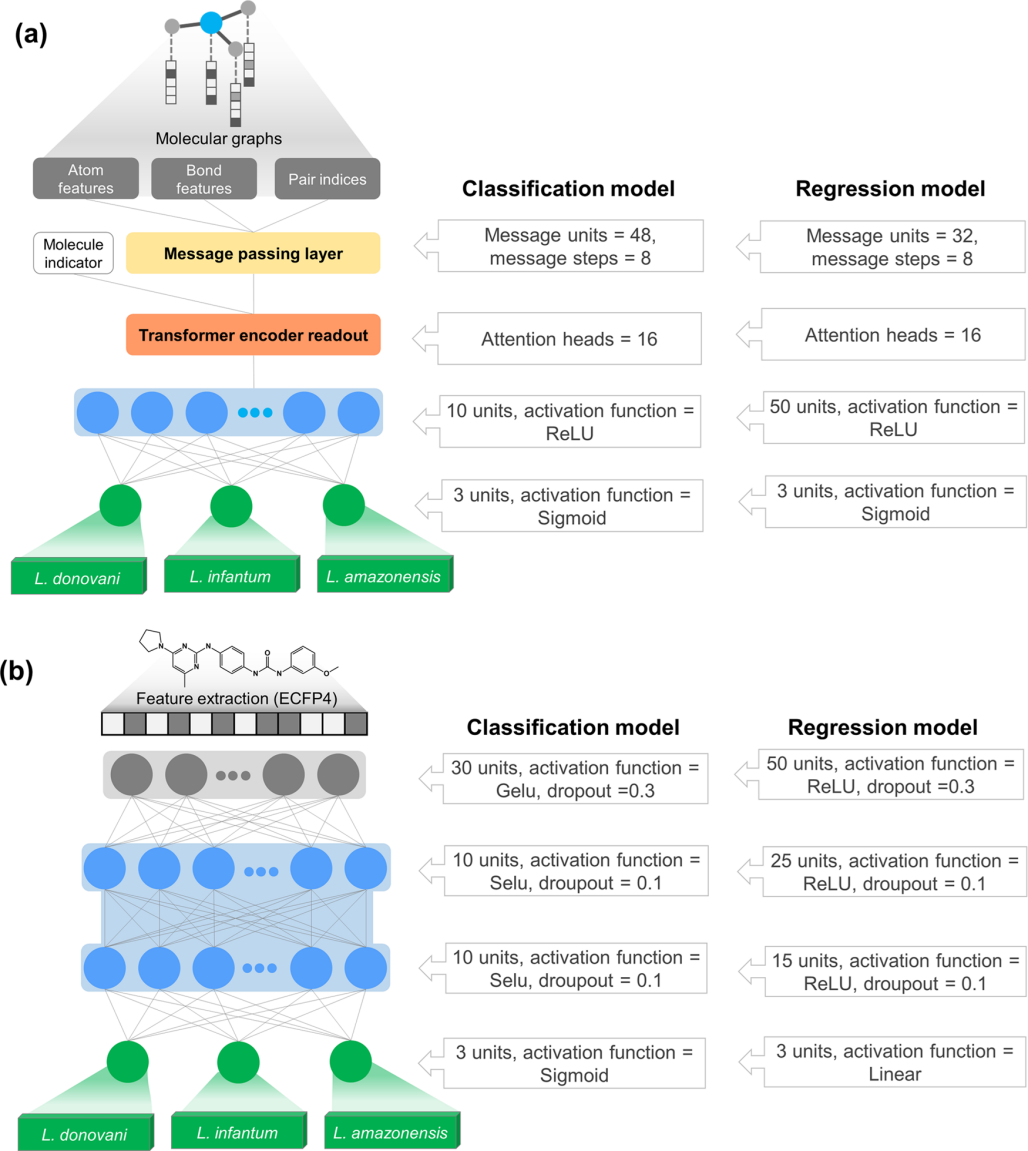
Schematic representation of the (a) MT-MPNN and (b) MT-DNN architectures and corresponding hyperparameters for the classification and regression models explored in this study.

Multitask learning models were developed using MPNN and DNN architectures to predict the antileishmanial activity of compounds across three *Leishmania* species. This approach is particularly beneficial when data is limited, as it leverages information from related tasks to improve generalization. In this study, these models enhanced knowledge transfer across species, improving the predictive power for the *L. infantum* task. As shown in Figure 3a, MT-MPNNs were designed to process molecular graphs comprehensively. They begin with input layers for atom features, bond features, and pair indices, capturing essential atomic and bond information. These features are then passed through a series of message-passing layers, which iteratively update node representations by aggregating messages from neighboring nodes and integrating bond and atom attributes. The updated node features are subsequently processed by a transformer encoder readout, which refines the representations through self-attention mechanisms, enhancing the model’s ability to capture complex interactions within the molecular graph. Finally, the processed features are fed into dense layers, culminating in the prediction of antileishmanial activities. Moreover, MT-DNNs were developed using multiple dense layers with hard-parameter sharing to prevent overfitting (see Figure 3b).^39^ This approach ensures shared parameters across tasks, leading to a more generalized feature learning. This design leverages the synergistic learning of multiple tasks, enhancing the predictive performance and robustness of the model in predicting compound activities against different species of *Leishmania*.

#### 3.3.1 Multitask Learning Performance

**Figure 4:**
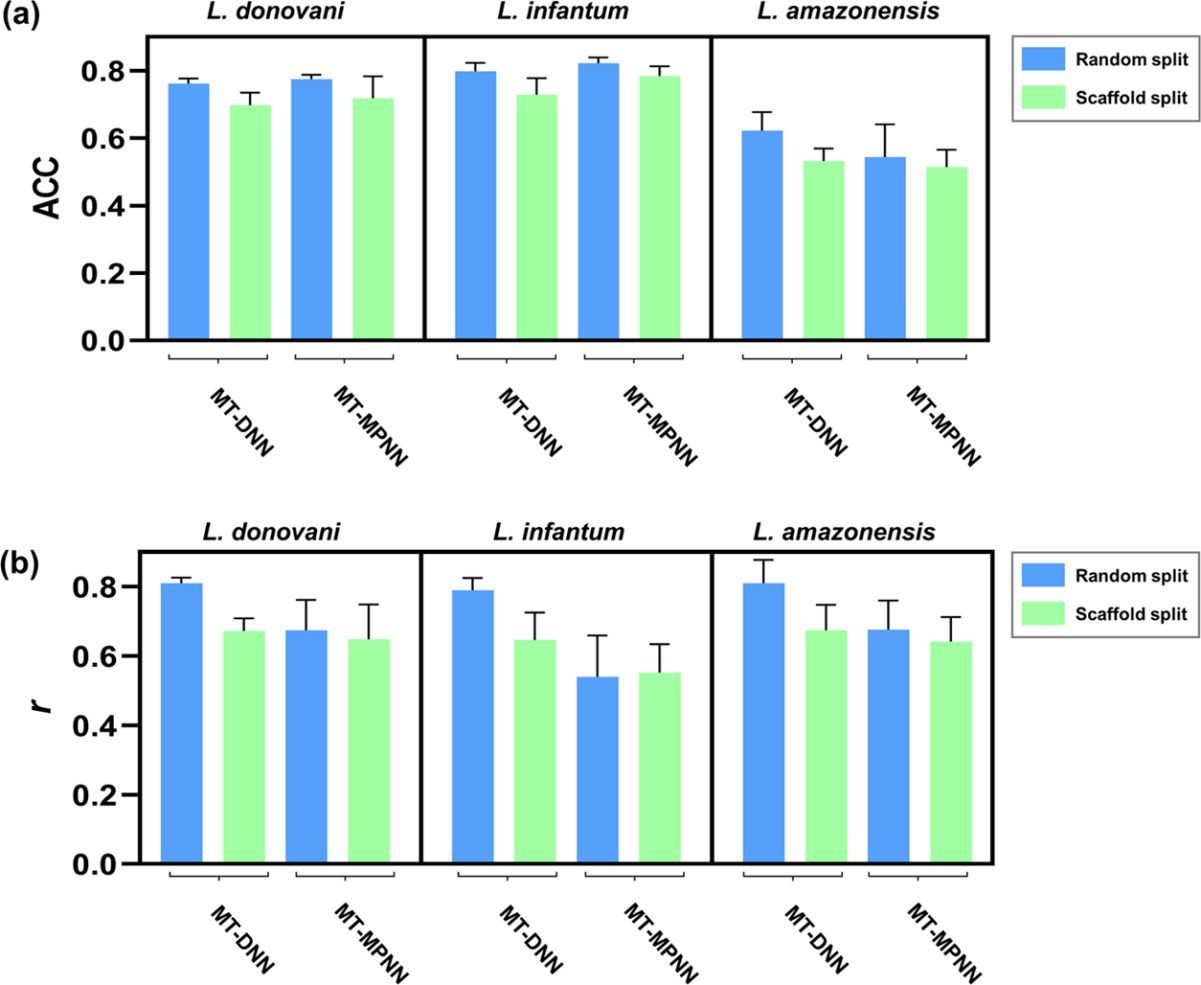
Test set performance of multitask (a) classification and (b) regression models developed using random and scaffold splits.

The test set performances of the MT-MPNN and MT-DNN classification and regression models, developed using a random split strategy, are detailed in Tables S1 and S2, respectively. The MT-MPNN classification model (Figure 4a) showed satisfactory test set ACC for *L. donovani* (0.77 ± 0.06) and *L. infantum* (0.73 ± 0.05) but failed to classify *L. amazonensis* test set compounds (0.59 ± 0.01). The MT-DNN classification model (Figure 4a) exhibited similar test set accuracy for *L. donovani* (0.76 ± 0.01) but demonstrated improved generalization for *L. infantum* (0.80 ± 0.02) and *L. amazonensis* (0.67 ± 0.04). Although none of the tasks achieved perfect predictive power and the performance on smaller data sets was somewhat lower, the MT-DNN classification model significantly outperformed the MT-MPNN models. This indicates that the MT-DNN approach effectively identified meaningful ECFP4 fingerprints related to target end points during the learning process, highlighting its superior capability in generalizing across different tasks.

Similarly, the MT-MPNN regression model (Figure 4b) failed to promote the inductive transfer of knowledge for the *L. infantum* task (*r* = 0.48 ± 0.16). In contrast, the corresponding MT-DNN regression model (Figure 4b) showed high predictive power with *r* values of 0.81 ± 0.02, 0.83 ± 0.06, and 0.85 ± 0.08 for *L. donovani*, *L. infantum*, and *L. amazonensis*, respectively. The corresponding RMSE values are 0.49 ± 0.03, 0.45 ± 0.05, and 0.43 ± 0.04, respectively. Scatter plots of the predicted vs experimental pIC_50_ values for the training, validation, and test sets are presented in Figure S2.

In addition to the standard random split approach used to explore the predictive power of the multitask models, we also utilized scaffold splitting, which provides a more challenging and realistic assessment of the model performance (Figure 4). Scaffold splitting ensures that there is no overlap of Murcko scaffolds between the training, validation, and test sets, thereby testing the model’s ability to generalize to novel chemical structures. Our results demonstrate that the models developed using the random split strategy consistently outperformed those based on scaffold split, highlighting the increased difficulty and stringent nature of scaffold-based evaluation.^35,36^

### 3.4 Multitask vs Single-Task Models

In this study, we developed statistically robust MT-DNN classification and regression models to predict the antileishmanial activity of untested compounds across three *Leishmania* species, focusing on enhancing the predictive power for the *L. infantum* task. By integrating data from multiple tasks, multitask learning effectively increases the overall data set size, enabling the models to learn more comprehensive and generalized features. This approach leads to balanced performance for each task. It reduces the risk of overfitting, as the shared knowledge across tasks stabilizes the variations and uncertainties inherent in smaller data sets.^40^

**Figure 5:**
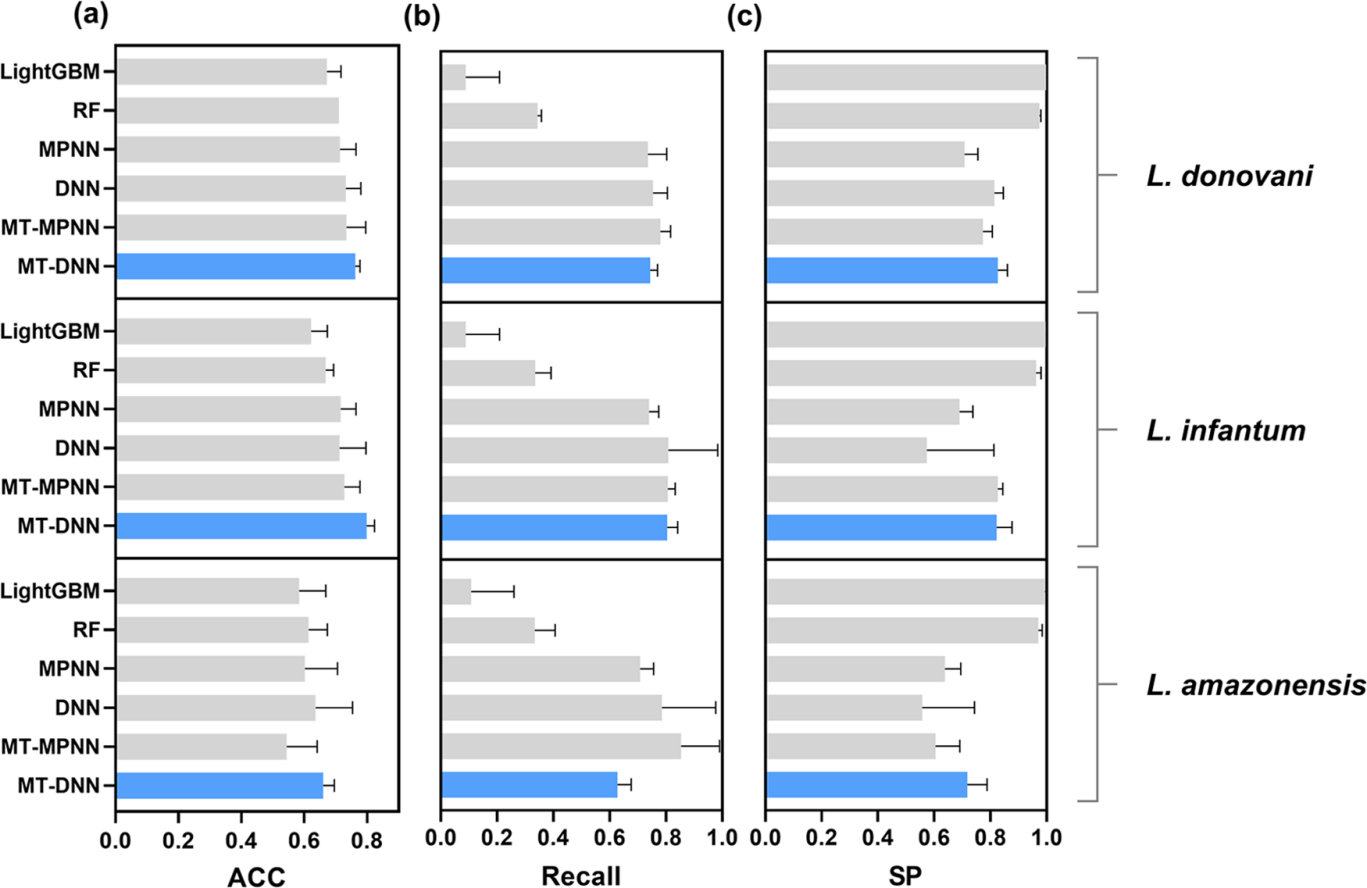
Average test set (a) ACC, (b) recall, and (c) SP of multitask and single-task classification models developed by using random splitting. The error bar represents the SD over five splitting runs.

We also employed a baseline analysis of MT-DNN models with single-task models to examine inductive knowledge transfer. Summarized accuracies, recall and specificities of the MT-DNN classification model and corresponding single-task classification models (i.e., DNN, MPNN, RF, and LightGBM) are shown in Figure 5. The detailed statistical characteristics of classification models are listed in Table S1. Overall, the MT-DNN classification model outperformed all single-task models regarding test set accuracies. In addition, this model is less sensitive to data imbalance, as indicated by consistent test set recall and specificities across all tasks. This reduced sensitivity to data imbalance highlights the robustness of the MT-DNN model in handling diverse and uneven data sets, making it a more reliable predictor of antileishmanial activity.

**Figure 6:**
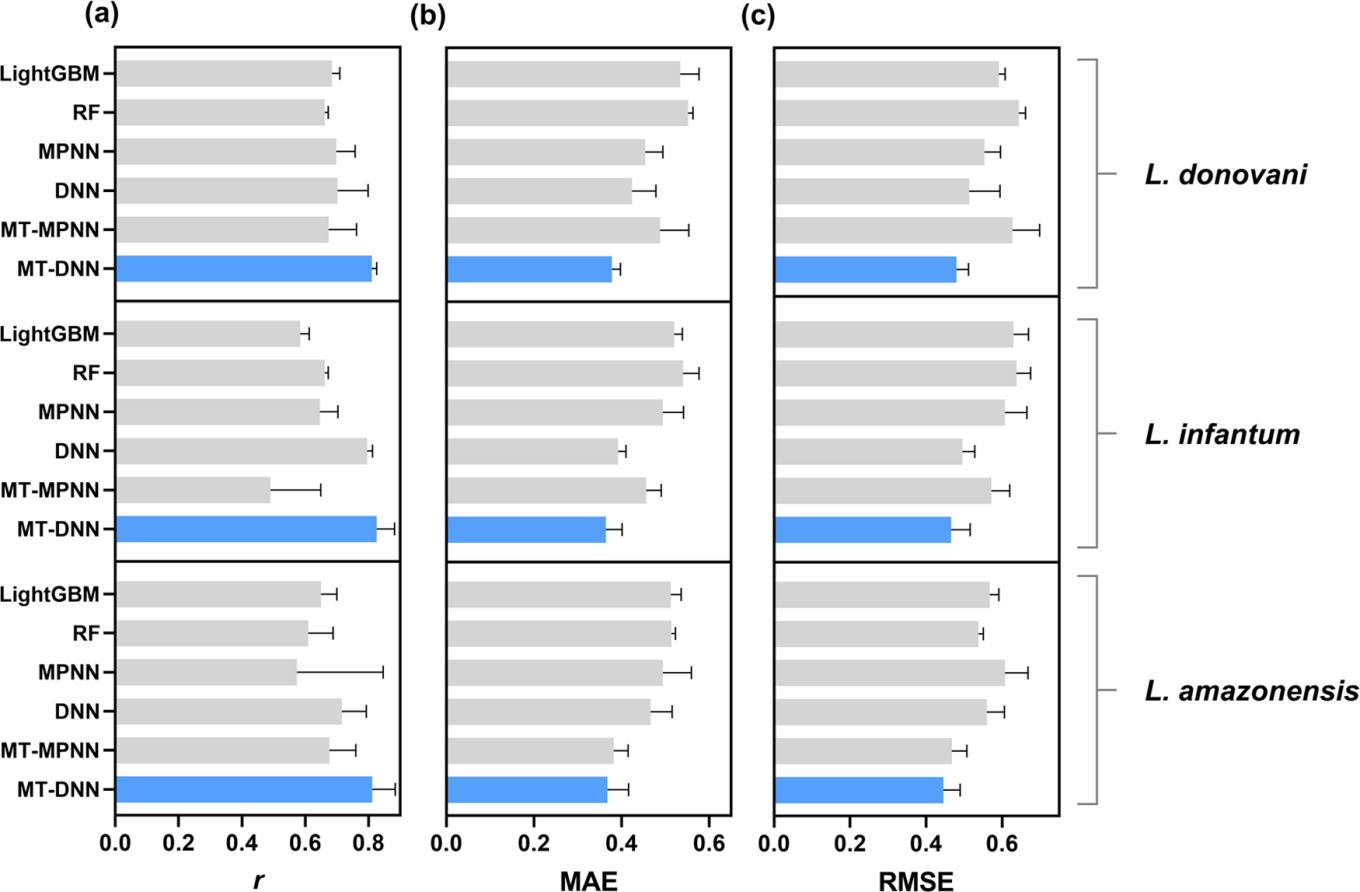
Average (a) *r*, (b) MAE, and (c) RMSE performances of multitask and single-task regression models developed by using random splitting. The error bar represents the SD over five splitting runs.

The performance comparison of the MT-DNN regression model against single-task models, as depicted in Figure 6 and Table S2, reveals that the multitasking approach consistently outperforms single-task models across all evaluation metrics. Specifically, the MT-DNN model presents the best *r* values among the baseline models, indicating stronger correlations between the predicted and experimental pIC_50_ values. Regarding error metrics, the MT-DNN model exhibited lower MAE and RMSE values across all models. Lower MAE values suggest that the predictions of the MT-DNN model are, on average, closer to the experimental values. Lower RMSE values indicate a better overall fit with fewer significant prediction errors. These results underscore the robustness of the MT-DNN regression model in predicting antileishmanial activity.

### 3.5 Structural Insights for the *L. infantum* Activity

**Figure 7:**
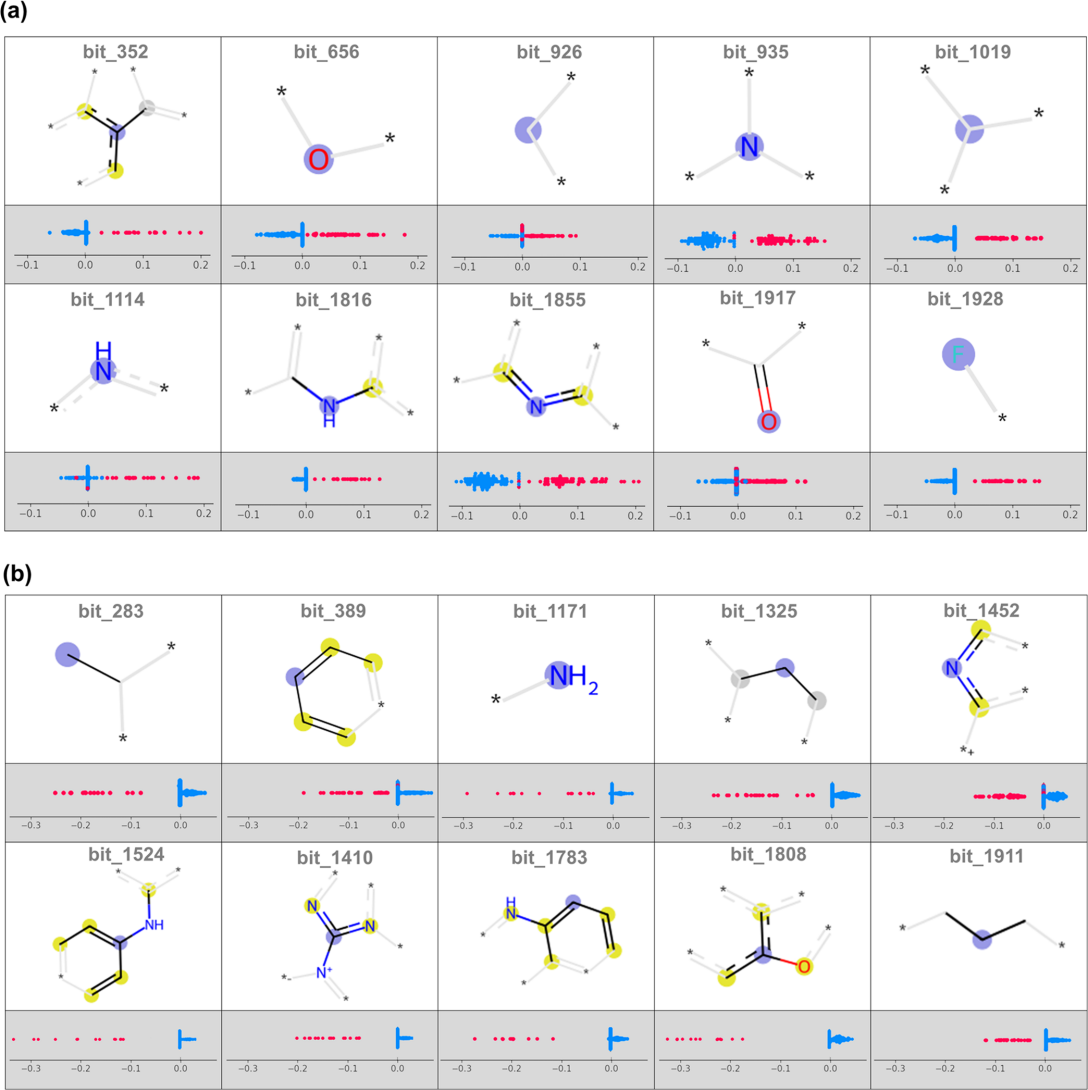
SHAP summary plot and global feature importance scores for ECFP4 features trained on the *L. infantum* task. Panel (a) represents features with positive contributions to activity, whereas panel (b) represents features with negative ones. The purple circle denotes the fingerprint’s center with a radius involving atoms denoted by yellow circles. The asterisk denotes a continuation of the structure. Each point represents a sample from the test set, where pink points indicate the presence of the fragment encoded by the bit, and blue points represent its absence.

A shortcoming of many deep learning approaches is the difficulty in rationalizing predictions. However, the lack of interpretability in these models can pose challenges for practitioners in medicinal chemistry, requiring transparency in cost-sensitive and time-sensitive scenarios such as hit-to-lead and lead optimization studies.^41−43^ To address this, concise and informative explanations for the *L. infantum* activity were provided using SHAP values derived from the MT-DNN regression model (Figure 7). These SHAP values assign a contribution score to each ECFP4 bit of the fingerprint vector, reflecting how much each contributes to the *L. infantum* activity.

The top 10 ECFP4 fragments with positive contributions to activity are shown in Figure 7a, highlighting structural motifs that consistently enhance predicted potency, such as nitrogen-containing heterocycles (bit_1114 and bit_1855, respectively), tertiary (bit_935) and secondary amines (bit_1816), and electronegative fragments such as carbonyl (bit_1917) and fluorine (bit_1928). These features are likely essential drivers of bioactivity against *L. infantum*. In contrast, Figure 7b highlights the ten fragments with the most negative contributions, identifying structural elements like hydrophobic groups (bit_283, bit_1325, and bit_1911), primary amines (bit_1171), simple aromatic systems (bit_389), positively charged nitrogen attached to a nitrogen-containing heterocycle (bit_1410), and bicyclic aromatic system containing oxygen (bit_1808) that reduce predicted activity. This global SHAP analysis offers a comprehensive understanding of the chemical features that positively or negatively affect activity against *L. infantum*, providing valuable insights to guide future compound optimization efforts.

### 3.6 Virtual Screening (VS)

**Figure 8:**
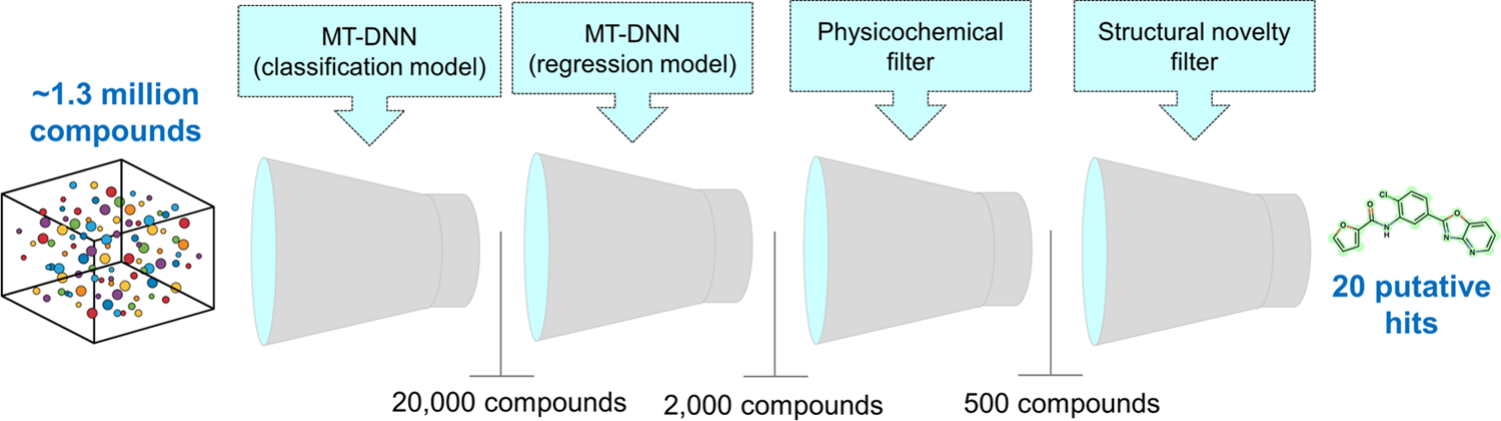
Virtual screening pipeline used to identify novel compounds against *L. infantum*. The physicochemical filter was based on aqueous solubility (cLogS > −5.0) and lipophilicity (cLogP between 0.5 and 4.0). A structural novelty filter was applied using Tanimoto similarity with ECFP4 fingerprints to ensure that the virtual hits had no prior experimental activity reported against *L. infantum*.

The MT-DNN models were employed as filters to screen putative compounds active against *L. infantum*, as outlined in Figure 8. Initially, the classification and regression models were used sequentially to screen approximately 1.3 million compounds from the CORE and EXPRESS-Pick collections of the ChemBridge database (http://www.chembridge.com). The classification model initially predicted 20,000 compounds as potential hits, from which the regression model further prioritized 2000 compounds with pIC_50_ > 5 (IC_50_ < 10 μM) against *L. infantum*. Subsequently, a physicochemical filter was applied to narrow down these candidates to 500 compounds, ensuring they had suitable aqueous solubility (cLogS > −5.0) and lipophilicity (cLogP between 0.5 and 4.0). At the final stage of the VS workflow, 20 putative hits were selected based on structural novelty and assessed using pairwise Tanimoto similarity analysis and visual inspection. Table 1 provides details of the chemical structures, physicochemical properties, and corresponding predictions of the prioritized compounds. These prioritized compounds were purchased for further experimental validation to confirm their *in vitro* efficacy against *L. infantum*. This multistep workflow ensured the identification of promising candidates with potential antileishmanial activity, advancing them toward experimental validation.

**Table 1:**
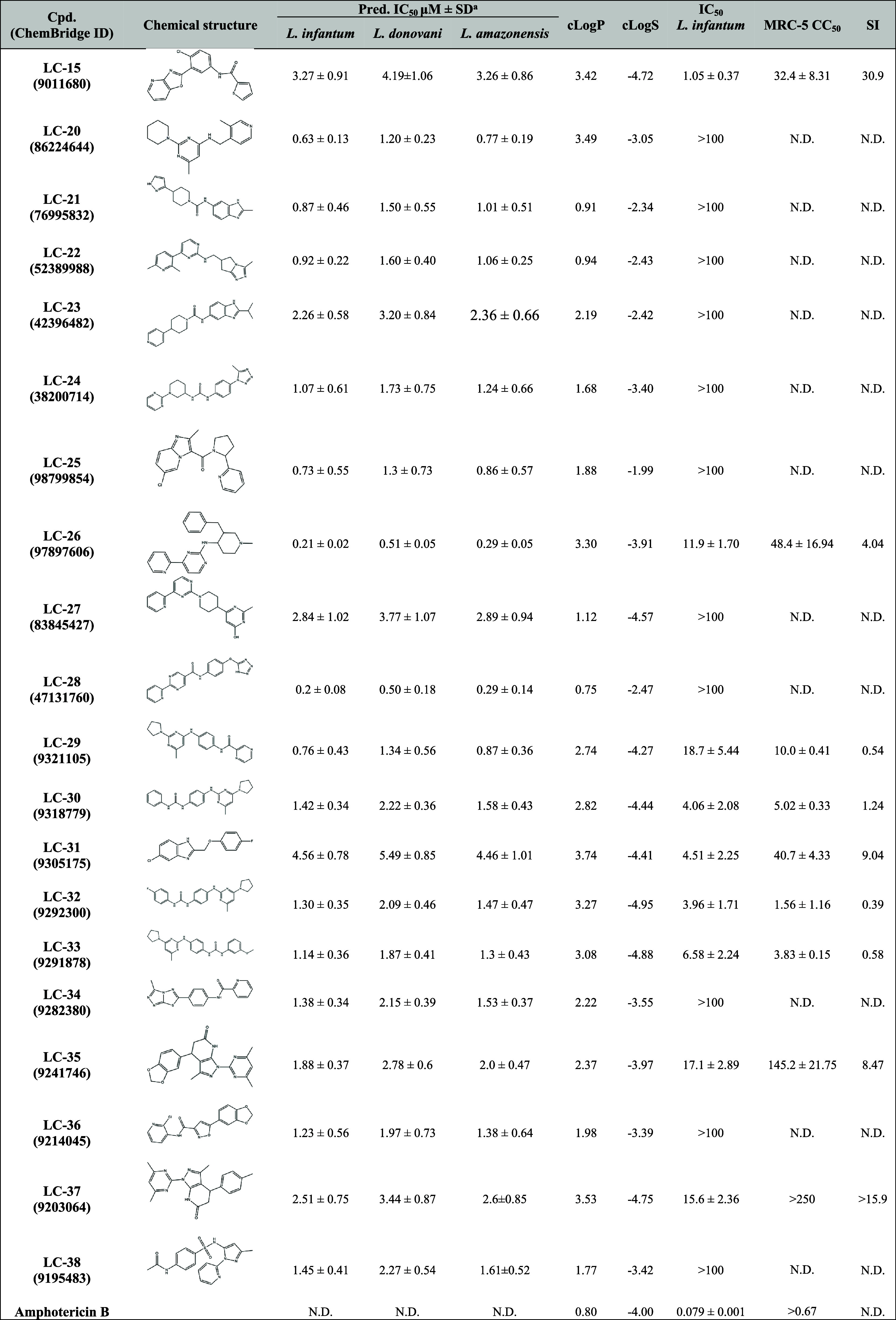
Predicted and Experimental Antileishmanial Activity and Cytotoxicity Data for the Prioritized Compounds

a The predicted values are the mean ± SD from five prediction runs; N.D.: not determined; LogS: log (solubility measured in mol/L); cLogP: predicted octanol–water partition coefficient; CC_50_: concentration required for the reduction of cell viability by 50% as compared to the untreated control; MRC-5: human fetal lung fibroblast cells; SI: selectivity index; AMPB: artesunate.

### 3.7 Experimental Validation

The 20 putative hits were evaluated *in vitro* against promastigotes of *L. infantum*. Among them, nine compounds (Table 1) exhibited significant antileishmanial activity, with IC_50_ values ranging from 1.05 to 18.7 μM. Notably, compounds **LC-15** (IC_50_ = 1.05 μM), **LC-26** (IC_50_ = 11.9 μM), **LC-29** (IC_50_ = 18.7 μM), **LC-30** (IC_50_ = 4.06 μM), **LC-31** (IC_50_ = 4.51 μM), **LC-32** (IC_50_ = 3.96 μM), **LC-33** (IC_50_ = 6.58 μM), **LC-35** (IC_50_ = 17.1 μM), and **LC-37** (IC_50_ = 15.6 μM) exhibited activity at low micromolar concentrations.

**Figure 9:**
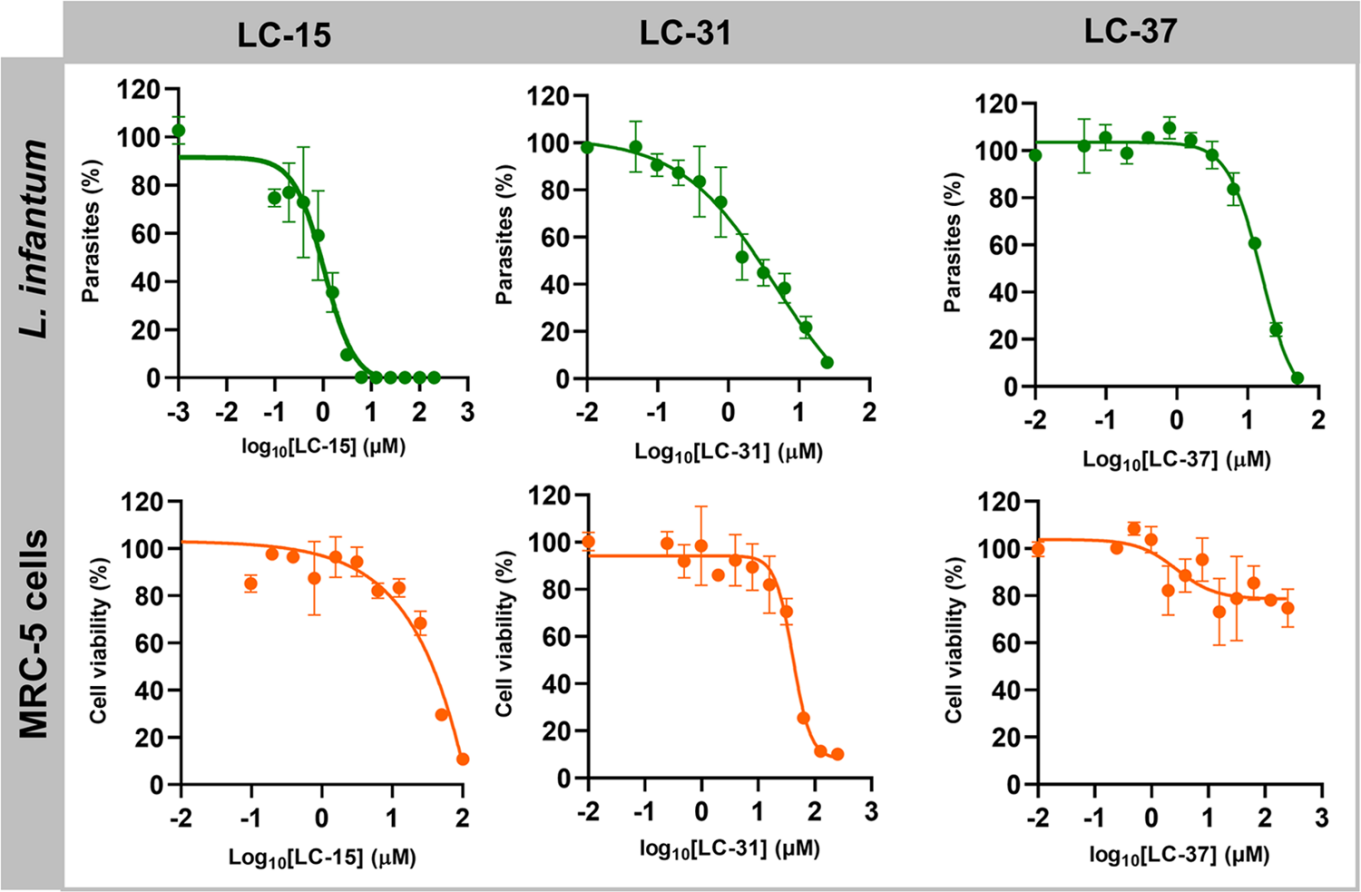
Dose–response curves depicting the antileishmanial activity and cytotoxicity of the top three hit compounds.

To assess the selectivity, cytotoxicity assays were performed on MRC-5 human fibroblast cells, and the selectivity index (SI) was calculated as the ratio of CC_50_ to IC_50_. As shown in Table 1, six compounds (**LC-26**, **LC-29**, **LC-30**, **LC-32**, **LC-33**, and **LC-35**) exhibited significant cytotoxicity toward MRC-5 cells, with SIs < 8.47, highlighting the challenge of maintaining selectivity. On the other hand, **LC-31** exhibited moderate cytotoxicity (CC_50_ = 40.7 μM, SI = 9.04), while **LC-37** demonstrated low cytotoxicity (CC_50_ > 250 μM, SI > 15.9). Overall, **LC-15** emerged as the most promising hit among the tested compounds, with a CC_50_ of 32.4 μM and an SI of 30.9. The dose–response curves of the top three hits are presented in Figure 9.

Interestingly, **LC-15** shares a high structural similarity with compound GNF5343, known for its antileishmanial activity.^44,45^ GNF5343 has been identified as a selective inhibitor of the *L. donovani* 20S proteasome, a critical target for parasite survival.^44,45^ Based on this structural similarity, **LC-15** is hypothesized to act as an *L. infantum* 20S proteasome inhibitor, a validated target for treating visceral leishmaniasis. According to Wyllie et al.,^45^ the nitrogen atom in the heterocyclic ring of these series is crucial for maintaining activity, as it engages in fundamental hydrogen bond interactions within the binding site of the 20S proteasome in kinetoplastid species. The amide group similarly plays an essential role by forming hydrogen bonds, further stabilizing the inhibitor’s interaction with the proteasome. This strengthens the hypothesis that **LC-15** acts as a 20S proteasome inhibitor, a validated target for antileishmanial therapy.^45^

**Figure 10:**
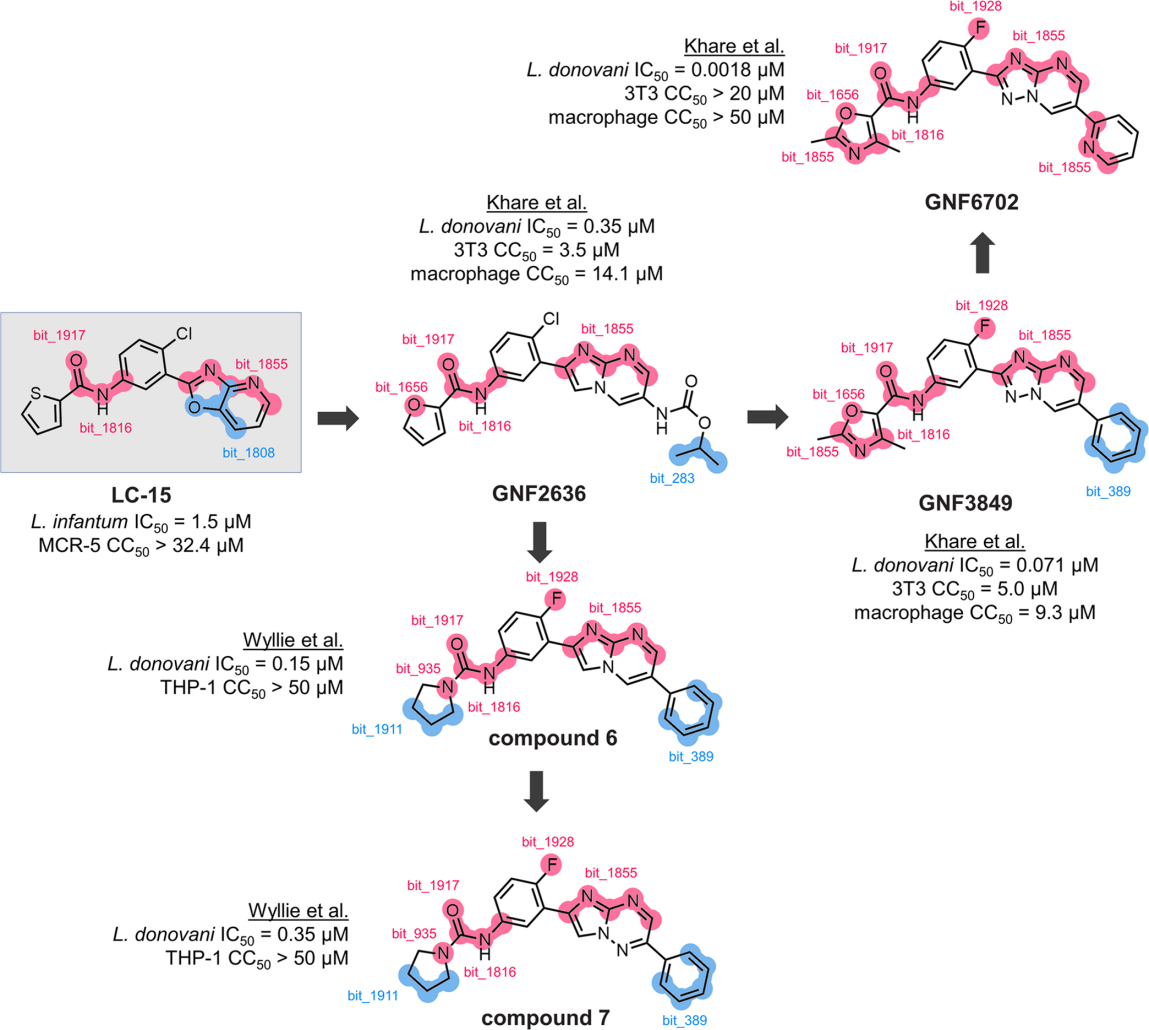
Structure–activity relationship of central ring substitutions in **LC-15** and known *L. donovani* 20S proteasome inhibitors with antileishmanial activity, utilizing global features prioritized through SHAP values. The bioassay data and chemical structures for the 20S proteasome inhibitors were retrieved from Wyllie et al.^45^ and Khare et al.^44^ Fragments highlighted in pink represent ECFP4 bits contributing positively to antileishmanial activity, while fragments shown in blue indicate features with negative contributions.

According to structure–activity relationship (SAR) analyses established in Figure 10, modifications to the [1,3]oxazolo[4,5-*b*]pyridine of **LC-15** can be achieved by replacing it with other heterocycles such as imidazo[1,2-*a*]pyrimidine (see GNF2636),^44^ [1,2,4]triazolo[1,5-*a*]pyrimidine (see GNF6702),^44^ or imidazo[1,2-*b*][1,2,4]triazine (see compound 7).^45^ These substitutions are anticipated to either retain or enhance the compound’s interaction within the binding site of the 20S proteasome by maintaining hydrogen bonding capacity and steric compatibility with the binding pocket. Replacing the thiophene in **LC-15** with other five-membered heterocycles (e.g., furan and 1,3-oxazole) and aliphatic rings (e.g., pyrrolidine) enhances potency, which is explained by the additional hydrogen bond formed in the proteasome’s active site. Furthermore, replacing the chlorine substituent on the phenyl ring with fluorine improves the antileishmanial potency. The fluorine atom is known to establish an orthogonal multipolar interaction and weak hydrogen bond in the binding site of the 20S proteasome.^45^ These substitutions align with the model’s predictions, as SHAP global feature analysis identifies these positions as significant contributors to activity, demonstrating consistency with well-established SAR principles. Overall, these findings underscore the potential of the multitask learning models developed in this study to establish a solid foundation for future hit-to-lead optimization efforts.

## 4 Conclusions

Developing models to predict the antileishmanial activity of untested compounds is difficult due to limited data availability, which affects the model’s reliability and robustness. This study focused on developing and validating MT-DNN models to accurately prioritize perspective antileishmanial hits targeting *L. infantum*. The main advantage of this strategy is its capacity to enhance predictions for tasks with a scarcity of data, such as *L. infantum*, by efficiently exchanging knowledge with tasks with a more significant amount of data. Consequently, the MT-DNN models developed in this study improved the generalizability of *L. infantum* predictions by leveraging shared information across related tasks through inductive transfer learning. Furthermore, the explainability of predictions revealed key molecular features for antileishmanial activity, aligning with established SAR principles. These models also allowed the identification of 20 putative hits, nine of which showed *in vitro* antileishmanial activity against *L. infantum* promastigotes in low micromolar ranges. This corresponds to a hit rate of 45%. Among them, three compounds exhibited notable potencies (IC_50_ of 1.05 to 15.6 μM) and moderate cytotoxicities (CC_50_ of 32.4 to >175 μM), positioning it as a promising candidate for further hit-to-lead optimization. In summary, the multitask learning framework described in this study is a reliable and easily understandable approach to enhancing the accuracy of identifying potential compounds that target *L. infantum*.

The results of the study can be summarized as follows:

- Explainable multitask learning models were employed to predict compound profiles against three *Leishmania* species simultaneously.
- MT-DNN models improved the generalizability of *L. infantum* predictions by leveraging shared information across related tasks.
- Model explainability revealed key molecular features for antileishmanial activity, aligning with established SAR principles.
- The MT-DNN models allowed the discovery of three new hits with potent antileishmanial activity and SI greater than 9-fold for MRC-5 cells.
- **LC-15** showed the most promising results with IC_50_ values of 1.05 μM and SI of 30.9 fold.
- Discovered compounds are promising candidates for prospective hit-to-led investigations.

## Data Availability

To ensure the reproducibility of our study, all of the codes and data sets are available on GitHub: https://github.com/LCi-UFG/Multitask-Learning.

## Supporting Information Available

Detailed description of statistical performance and spectral data of test compounds (PDF)
