# Supplementary material for: Deep Multitask Learning-Driven Discovery of New Compounds Targeting Leishmania infantum
Source: ACS Omega. 2024 Dec 16;9(52):51271–84. doi: 10.1021/acsomega.4c07994 (PMC11696749; doi:10.1021/acsomega.4c07994)
Supplement: Supplementary file 1 — ao4c07994_si_001.pdf [file ao4c07994_si_001.pdf]

Deep Multitask Learning-Driven Discovery of New Compounds Targeting *Leishmania infantum*

Eder Soares de Almeida Santos ‡,[1], Jade Milhomem Lemos ‡,[1], Alexandra Maria dos Santos Carvalho ‡,[2], Felipe da Silva Mendonça de Melo[2], Eufrasia de Sousa Pereira[1], José Teófilo Moreira-Filho[3], Rodolpho de Campos Braga[3], Eugene N. Muratov[4], Philippe Grellier[5], Sébastien Charneau[6], Izabela Marques Dourado Bastos[2], Bruno Junior Neves[1,*]

[1] Laboratory of Cheminformatics, Faculty of Pharmacy, Federal University of Goiás, Goiânia, Brazil

[2] Pathogen-Host Interface Laboratory, Department of Cell Biology, Institute of Biological Sciences, University of Brasilia, Brasilia, Brazil

[3] InsilicAll Ltda, São Paulo, Brazil

[4] Laboratory for Molecular Modeling, UNC Eshelman School of Pharmacy, The University of North Carolina at Chapel Hill, North Carolina, USA

[5] UMR 7245 Molécules de Communication et Adaptation des Micro-organismes, Muséum National d'Histoire Naturelle, Équipe Parasites et Protistes Libres, Paris, France

[6] Laboratory of Protein Chemistry and Biochemistry, Department of Cell Biology, Institute of Biological Sciences, University of Brasilia, Brasilia, Brazil

*Author for correspondence: brunoneves@ufg.br

‡ Authors contributed equally

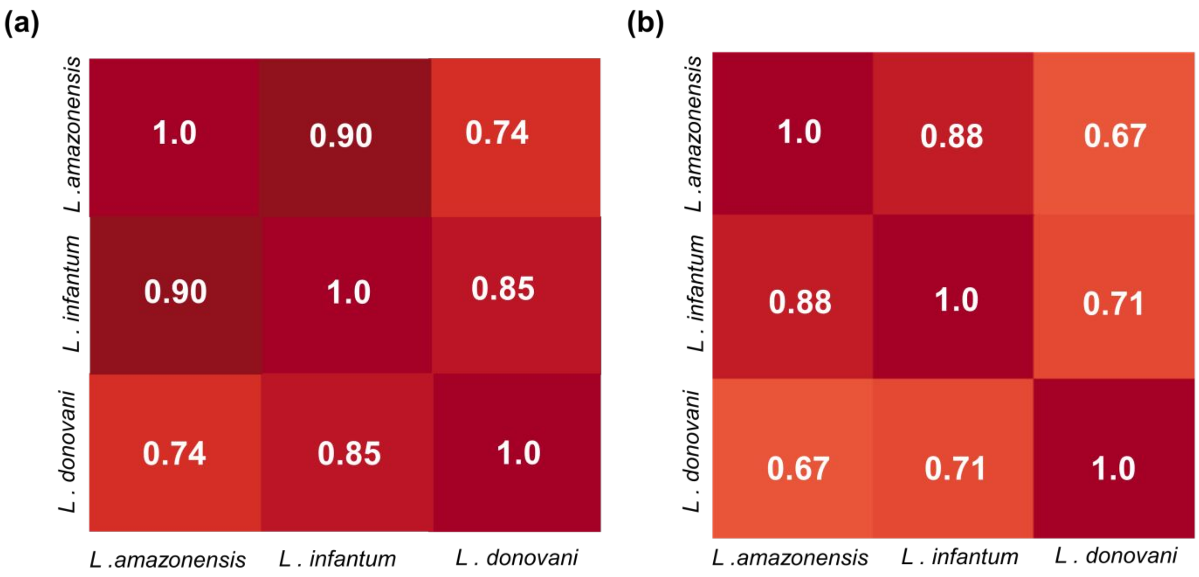

Figure S1. Heat maps showing the concordance among activity outcomes (**a**) and correlation among the pIC_{50} values (**b**) of the *L. donovani*, *L. infantum*, and *L. amazonensis* tasks.

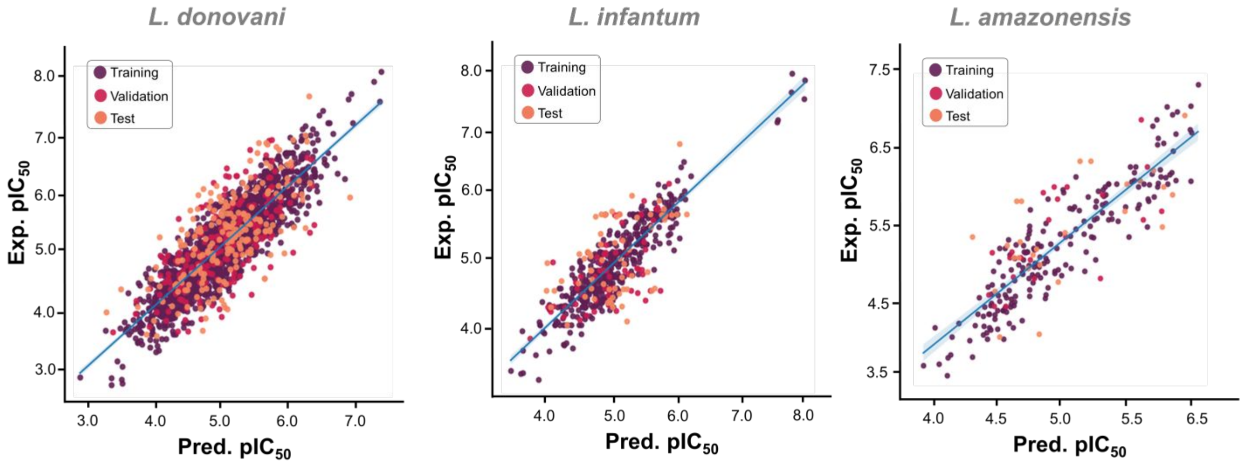

Figure S2. Scatter plots of predicted vs. experimental pIC_{50} values for the *L. donovani*, *L. infantum*, and *L. amazonensis* tasks of the MT-DNN regression model.

Table S1. Summarized statistical characteristics of multitask and single-task classification models.

Split	Set	*L. amazonensis*				*L. infantum*				*L. donovani*			
		ACC	Recall	SP	MCC	ACC	Recall	SP	MCC	ACC	Recall	SP	MCC
**Multitask Deep Neural Network (MT-DNN)**													
**8:1:1**	Training	0.85 ± 0.10	0.86 ± 0.05	0.79 ± 0.11	0.62 ± 0.07	0.87 ± 0.02	0.93 ± 0.02	0.91 ± 0.04	0.66 ± 0.06	0.92 ± 0.02	0.90 ± 0.02	0.95 ± 0.01	0.88 ± 0.02
	Validation	0.76 ± 0.05	0.62 ± 0.04	0.74 ± 0.06	0.53 ± 0.06	0.80 ± 0.03	0.81 ± 0.03	0.83 ± 0.05	0.53 ± 0.07	0.79 ± 0.03	0.75 ± 0.02	0.82 ± 0.03	0.53 ± 0.05
	Test	0.67 ± 0.04	0.62 ± 0.05	0.75 ± 0.07	0.49 ± 0.05	0.80 ± 0.02	0.81 ± 0.04	0.83 ± 0.05	0.55 ± 0.05	0.76 ± 0.01	0.75 ± 0.03	0.84 ± 0.03	0.52 ± 0.03
**Multitask Message-Passing Neural Network (MT-MPNN)**													
**8:1:1**	Training	0.82 ± 0.06	0.94 ± 0.08	0.68 ± 0.09	0.55 ± 0.09	0.9 ± 0.01	0.9 ± 0.01	0.89 ± 0.02	0.78 ± 0.02	0.89 ± 0.02	0.93 ± 0.01	0.88 ± 0.02	0.76 ± 0.03
	Validation	0.67 ± 0.04	0.80 ± 0.14	0.61 ± 0.09	0.42 ± 0.07	0.8 ± 0.02	0.8 ± 0.03	0.82 ± 0.02	0.54 ± 0.02	0.77 ± 0.06	0.79 ± 0.04	0.78 ± 0.03	0.56 ± 0.01
	Test	0.59 ± 0.01	0.83 ± 0.14	0.61 ± 0.09	0.48 ± 0.09	0.73 ± 0.05	0.79 ± 0.02	0.8 ± 0.02	0.57 ± 0.02	0.77 ± 0.06	0.80 ± 0.04	0.78 ± 0.03	0.61 ± 0.06
**Deep Neural Network (DNN)**													
**8:1:1**	Training	0.97 ± 0.06	0.99 ± 0.10	0.98 ± 0.08	0.94 ± 0.12	0.96 ± 0.01	0.97 ± 0.01	0.96 ± 0.01	0.92 ± 0.02	0.88 ± 0.1	0.99 ± 0.01	0.83 ± 0.14	0.76 ± 0.19
	Validation	0.68 ± 0.08	0.69 ± 0.17	0.64 ± 0.05	0.35 ± 0.14	0.77 ± 0.02	0.71 ± 0.06	0.79 ± 0.03	0.52 ± 0.04	0.65 ± 0.08	0.71 ± 0.17	0.63 ± 0.22	0.27 ± 0.13
	Test	0.59 ± 0.12	0.80 ± 0.19	0.52 ± 0.19	0.34 ± 0.26	0.75 ± 0.08	0.90 ± 0.18	0.61 ± 0.24	0.55 ± 0.02	0.75 ± 0.05	0.75 ± 0.05	0.81 ± 0.03	0.37 ± 0.10
**Message-Passing Neural Network (MPNN)**													
**8:1:1**	Training	0.70 ± 0.10	0.96 ± 0.03	0.65 ± 0.09	0.43 ± 0.02	0.89 ± 0.01	0.89 ± 0.01	0.90 ± 0.01	0.77 ± 0.01	0.88 ± 0.01	0.93 ± 0.01	0.86 ± 0.02	0.74 ± 0.03
	Validation	0.64 ± 0.08	0.83 ± 0.09	0.60 ± 0.08	0.47 ± 0.02	0.79 ± 0.01	0.78 ± 0.01	0. 79 ± 0.01	0.56 ± 0.02	0.83 ± 0.03	0.84 ± 0.03	0.82 ± 0.03	0.59 ± 0.06
	Test	0.65 ± 0.10	0.71 ± 0.05	0.64 ± 0.03	0.41 ± 0.01	0.74 ± 0.05	0.74 ± 0.03	0.69 ± 0.05	0.53 ± 0.02	0.74 ± 0.05	0.76 ± 0.07	0.70 ± 0.05	0.55 ± 0.01
**Random Forest (RF)**													
**5FCV**	Training	0.66 ± 0.01	0.28 ± 0.03	0.98 ± 0.01	0.37 ± 0.01	0.71 ± 0.01	0.32 ± 0.01	0.98 ± 0.01	0.40 ± 0.01	0.66 ± 0.02	0.27 ± 0.07	0.98 ± 0.02	0.38 ± 0.05
	Test	0.65 ± 0.06	0.32 ± 0.07	0.98 ± 0.01	0.33 ± 0.06	0.66 ± 0.02	0.34 ± 0.06	0.97 ± 0.02	0.38 ± 0.06	0.71 ± 0.00	0.35 ± 0.01	0.97 ± 0.01	0.34 ± 0.02
**Light Gradient Boosting Machine (LightGBM)**													
**5FCV**	Training	0.69 ± 0.02	0.24 ± 0.01	0.98 ± 0.05	0.29 ± 0.04	0.76 ± 0.01	0.25 ± 0.01	0.96 ± 0.02	0.33 ± 0.01	0.68± 0.01	0.21 ± 0.01	0.99 ± 0.02	0.41 ± 0.02
	Test	0.58 ± 0.08	0.01 ± 0.15	1.00 ± 0.01	0.37 ± 0.02	0.63 ± 0.05	0.01 ± 0.11	1.00 ± 0.01	0.27 ± 0.10	0.65 ± 0.04	0.01 ± 0.12	1.00 ± 0.00	0.34 ± 0.07

Table S2. Summarized statistical characteristics of multitask and single-task regression models.

Split	Set	*L. amazonenses*			*L. infantum*			*L. donovani*		
		r	**RMSE**	**MAE**	***r***	**RMSE**	**MAE**	***r***	**RMSE**	**MAE**
**Multitask Deep Neural Network (MT-DNN)**										
**8:1:1**	Training	0.92 ± 0.02	0.28 ± 0.03	0.23 ± 0.02	0.97 ± 0.01	0.22 ± 0.01	0.17 ± 0.01	0.98 ± 0.01	0.19 ± 0.05	0.15 ± 0.04
	Validation	0.83 ± 0.07	0.43 ± 0.05	0.34 ± 0.05	0.81 ± 0.02	0.49 ± 0.03	0.38 ± 0.02	0.73 ± 0.04	0.49 ± 0.02	0.40 ± 0.01
	Test	0.85 ± 0.08	0.43 ± 0.04	0.34 ± 0.05	0.83 ± 0.06	0.45 ± 0.05	0.37 ± 0.04	0.81 ± 0.02	0.49 ± 0.03	0.38 ± 0.02
**Multitask Message-Passing Neural Network (MT-MPNN)**										
**8:1:1**	Training	0.73 ± 0.02	0.30 ± 0.07	0.46 ± 0.05	0.69 ± 0.09	0.54 ± 0.12	0.44 ± 0.09	0.88 ± 0.07	0.54 ± 0.11	0.44 ± 0.09
	Validation	0.59 ± 0.09	0.34 ± 0.12	0.49 ± 0.08	0.60 ± 0.08	0.36 ± 0.10	0.50 ± 0.06	0.60 ± 0.04	0.36 ± 0.05	0.48 ± 0.04
	Test	0.63 ± 0.08	0.48 ± 0.04	0.40 ± 0.03	0.48 ± 0.16	0.57 ± 0.05	0.45 ± 0.03	0.81 ± 0.02	0.49 ± 0.03	0.46 ± 0.07
**Deep Neural Network (DNN)**										
**8:1:1**	Training	0.73 ± 0.02	0.30 ± 0.07	0.46 ± 0.05	0.69 ± 0.09	0.54 ± 0.12	0.44 ± 0.09	0.88 ± 0.07	0.54 ± 0.11	0.44 ± 0.09
	Validation	0.59 ± 0.09	0.34 ± 0.12	0.49 ± 0.08	0.60 ± 0.08	0.36 ± 0.10	0.50 ± 0.06	0.60 ± 0.04	0.36 ± 0.05	0.48 ± 0.04
	Test	0.75 ± 0.08	0.57 ± 0.05	0.48 ± 0.05	0.80 ± 0.02	0.48 ± 0.03	0.38 ± 0.02	0.71 ± 0.10	0.50 ± 0.08	0.40 ± 0.05
**Message-Passing Neural Network (MPNN)**										
**8:1:1**	Training	0.84 ± 0.01	0.54 ± 0.01	0.33 ± 0.04	0.78 ± 0.02	0.60 ± 0.02	0.44 ± 0.04	0.85 ± 0.05	0.41 ± 0.01	0.35 ± 0.04
	Validation	0.62 ± 0.09	0.66 ± 0.02	0.47 ± 0.03	0.67 ± 0.04	0.62 ± 0.05	0.49 ± 0.01	0.69 ± 0.04	0.32 ± 0.05	0.43 ± 0.02
	Test	0.67 ± 0.27	0.63 ± 0.06	0.49 ± 0.07	0.65 ± 0.06	0.60 ± 0.06	0.47 ± 0.05	0.71 ± 0.10	0.56 ± 0.04	0.46 ± 0.04
**Random Forest (RF)**										
**5FCV**	Training	0.64 ± 0.02	0.53 ± 0.02	0.50 ± 0.01	0.64 ± 0.01	0.65 ± 0.01	0.56 ± 0.01	0.70 ± 0.00	0.63 ± 0.01	0.53 ± 0.00
	Test	0.58 ± 0.08	0.54 ± 0.01	0.52 ± 0.01	0.67 ± 0.01	0.63 ± 0.04	0.54 ± 0.04	0.67 ± 0.01	0.64 ± 0.02	0.55 ± 0.01
**Light Gradient Boosting Machine (LightGBM)**										
**5FCV**	Training	0.76 ± 0.09	0.49 ± 0.10	0.40 ± 0.10	0.77 ± 0.01	0.58 ± 0.01	0.50 ± 0.01	0.75 ± 0.01	0.58 ± 0.01	0.53 ± 0.00
	Test	0.66 ± 0.05	0.57 ± 0.02	0.51 ± 0.02	0.58 ± 0.03	0.61 ± 0.04	0.51 ± 0.02	0.68 ± 0.03	0.58 ± 0.02	0.52 ± 0.04

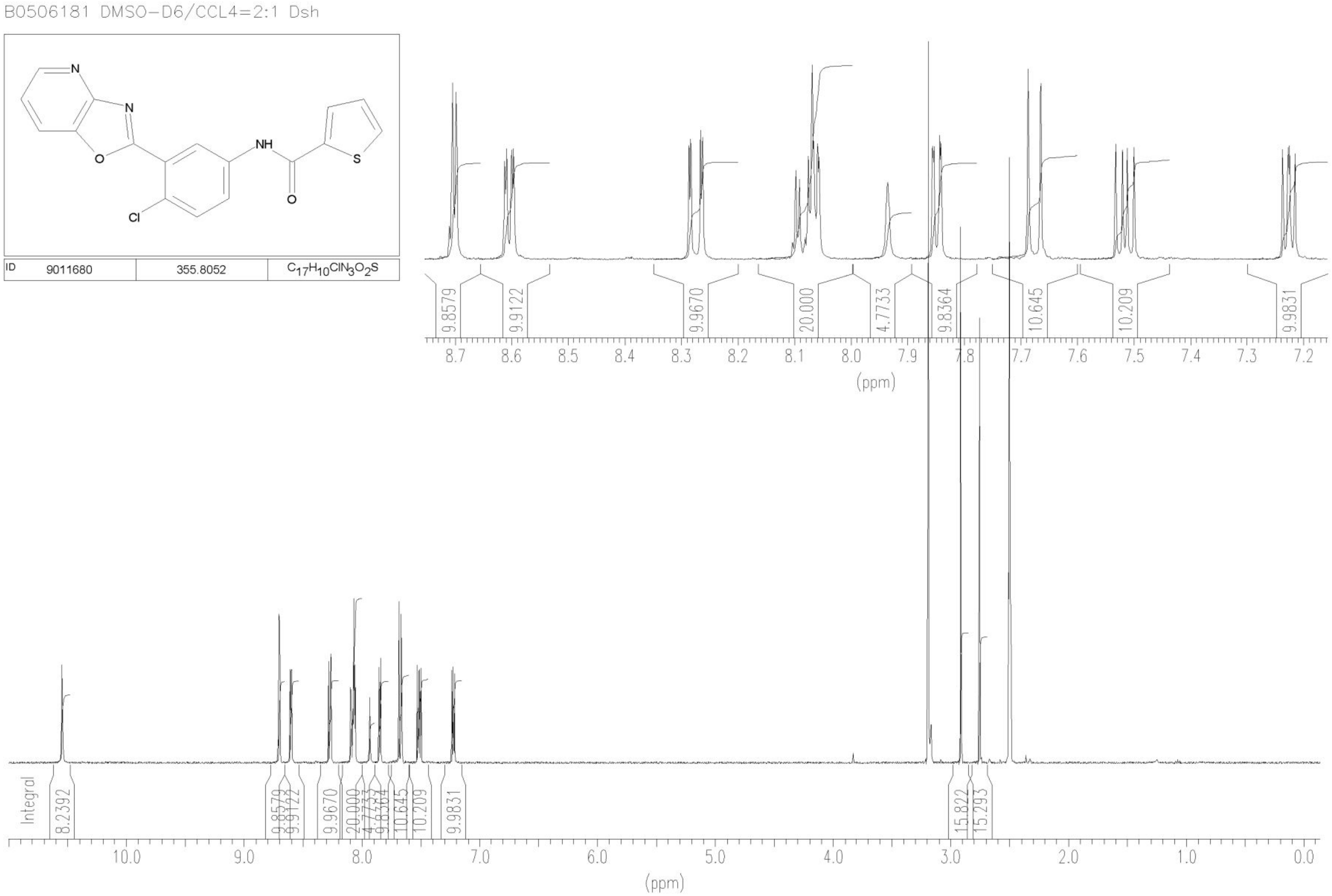

Figure S3. LC-15 (N-(4-chloro-3-[1,3]oxazolo[4,5-b]pyridin-2-ylphenyl)-2-thiophenecarboxamide).

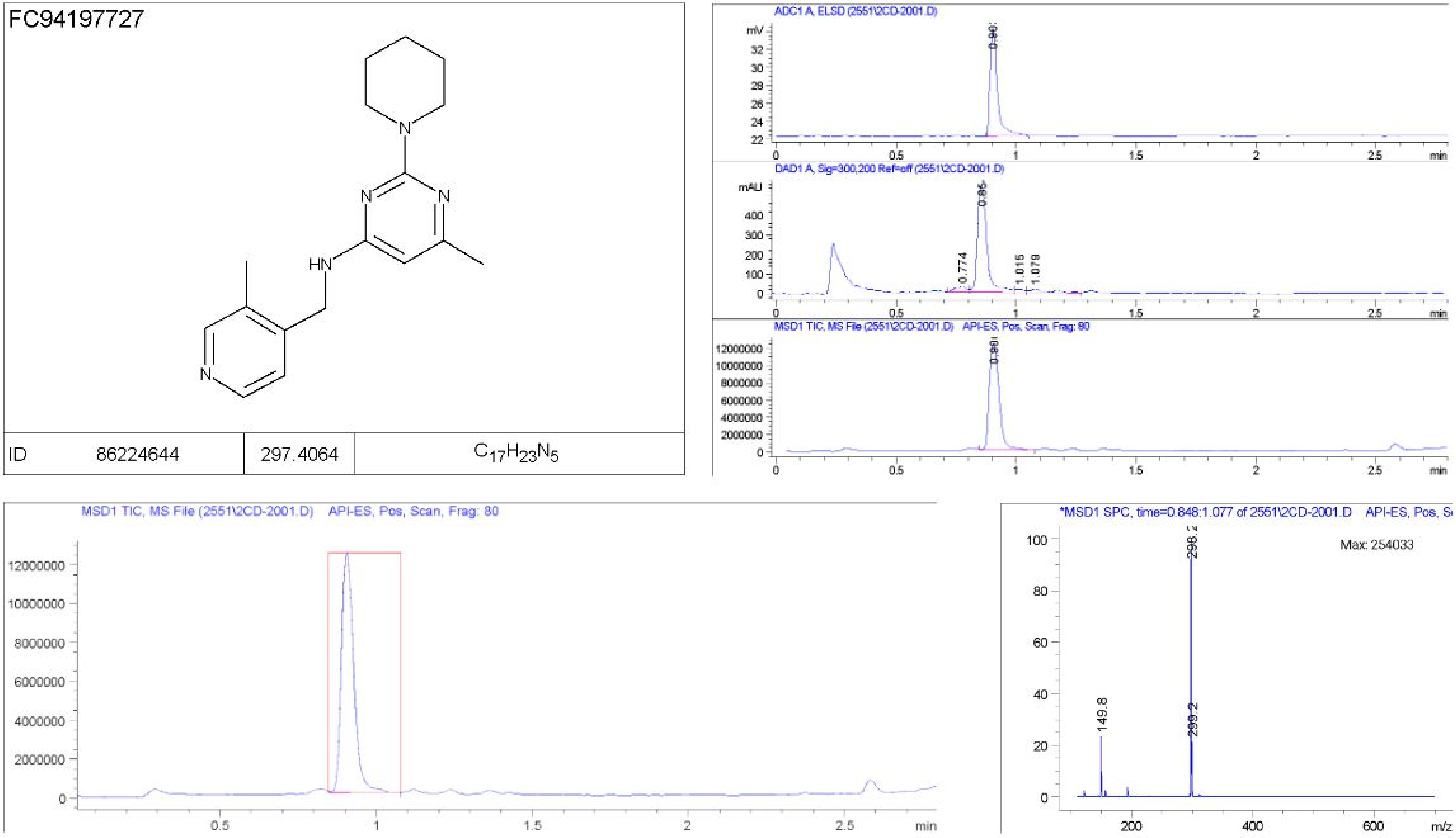

Figure S4. LC-20 (6-methyl-N-[(3-methylpyridin-4-yl)methyl]-2-piperidin-1-ylpyrimidin-4-amine).

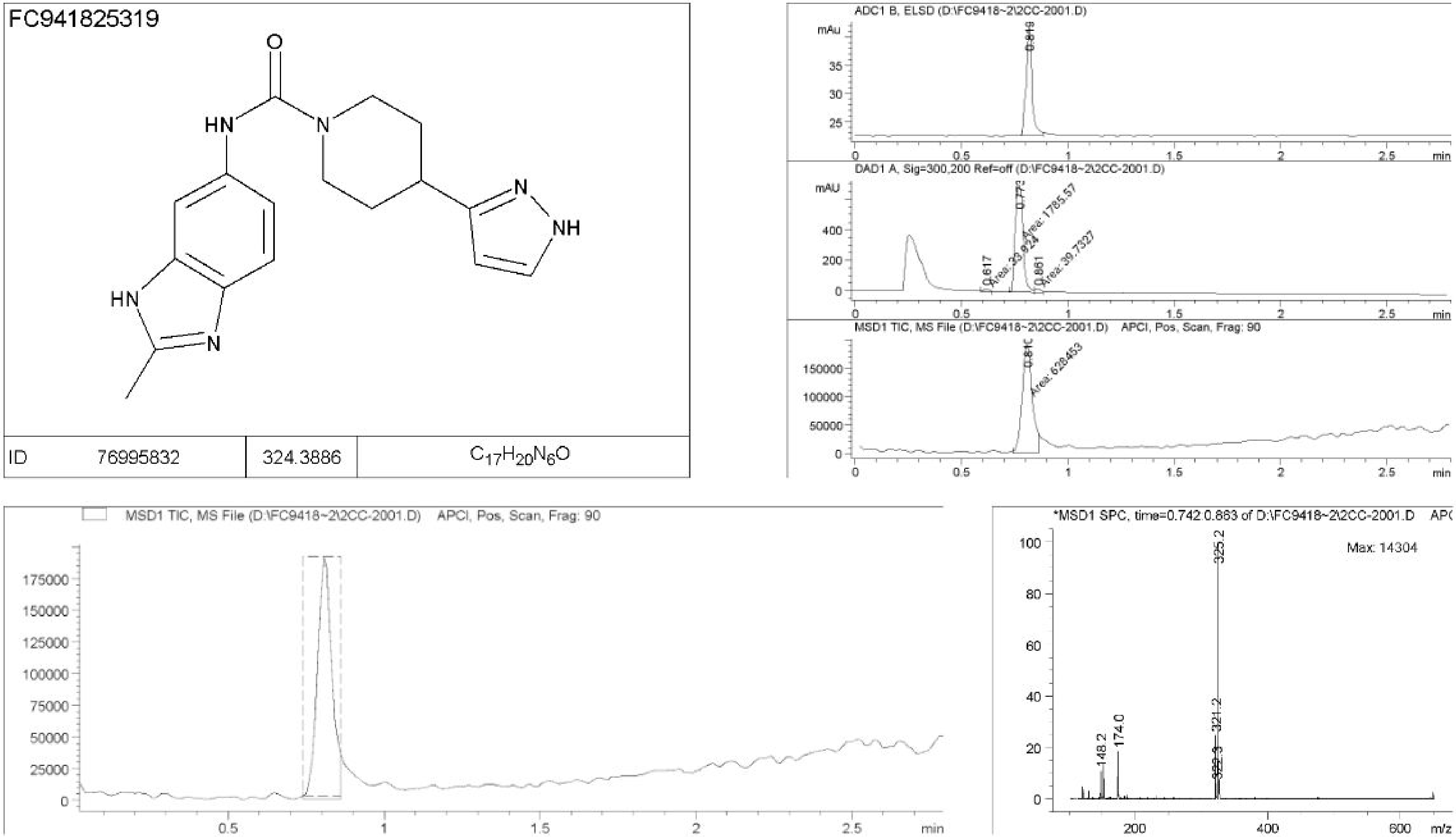

Figure S5. LC-21 (N-(2-methyl-1H-benzimidazol-6-yl)-4-(1H-pyrazol-3-yl)piperidine-1-carboxamide).

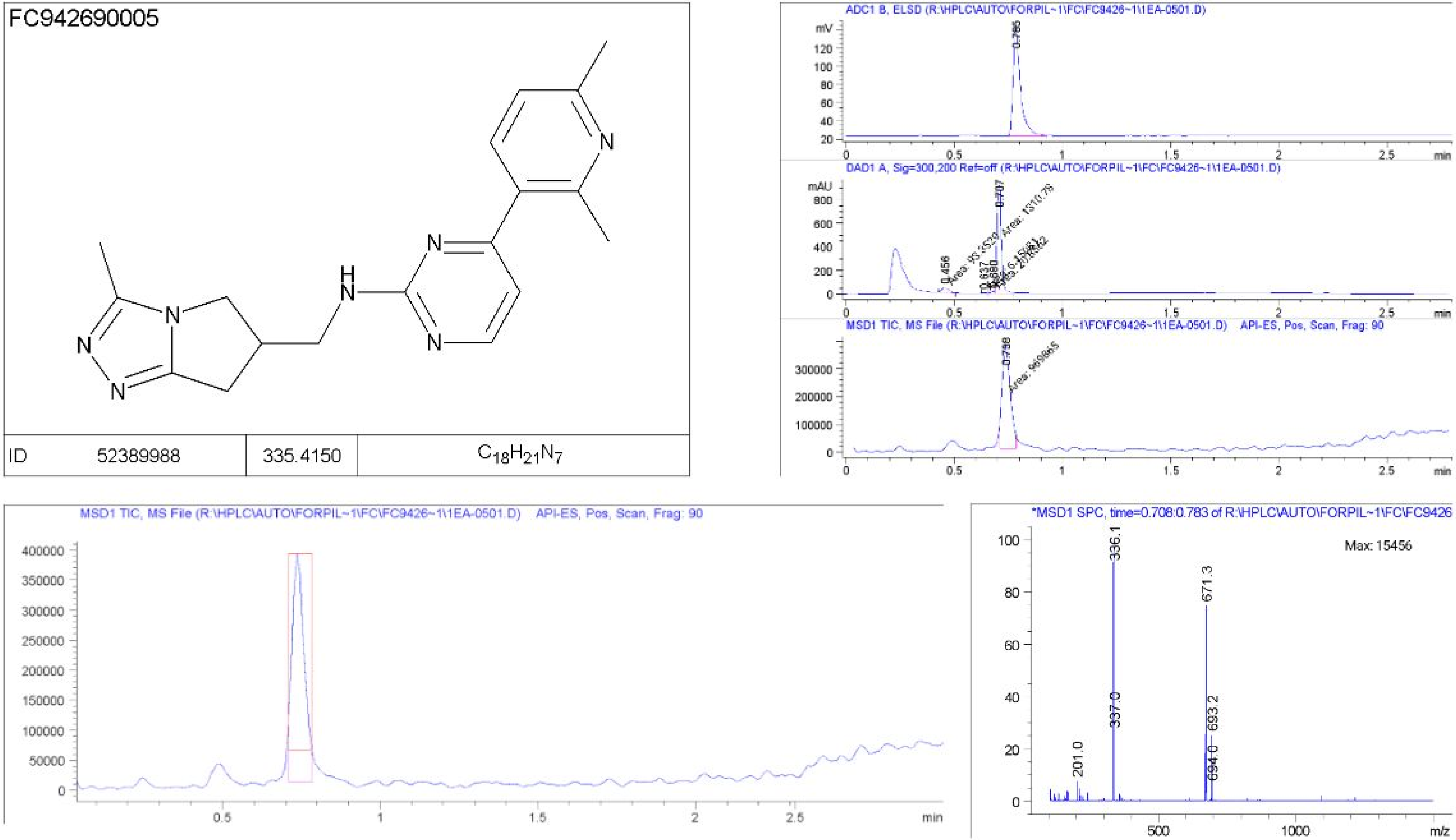

Figure S6. LC-22 (4-(2,6-dimethyl-3-pyridinyl)-N-[(3-methyl-6,7-dihydro-5H-pyrrolo[2,1-c][1,2,4]triazol-6-yl)methyl]-2-pyrimidinamine).

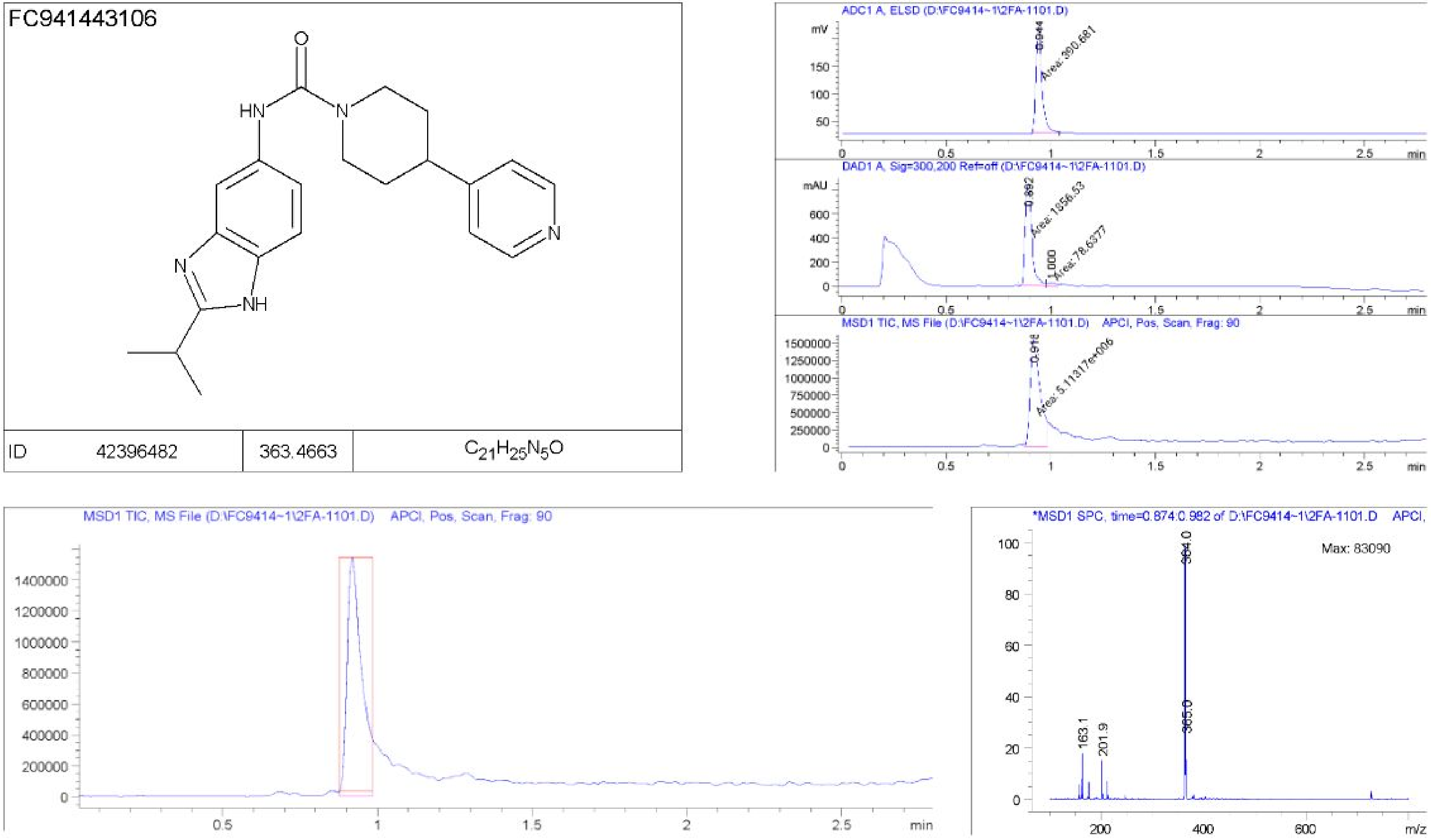

Figure S7. LC-23 (N-(2-isopropyl-1H-benzimidazol-5-yl)-4-pyridin-4-ylpiperidine-1-carboxamide).

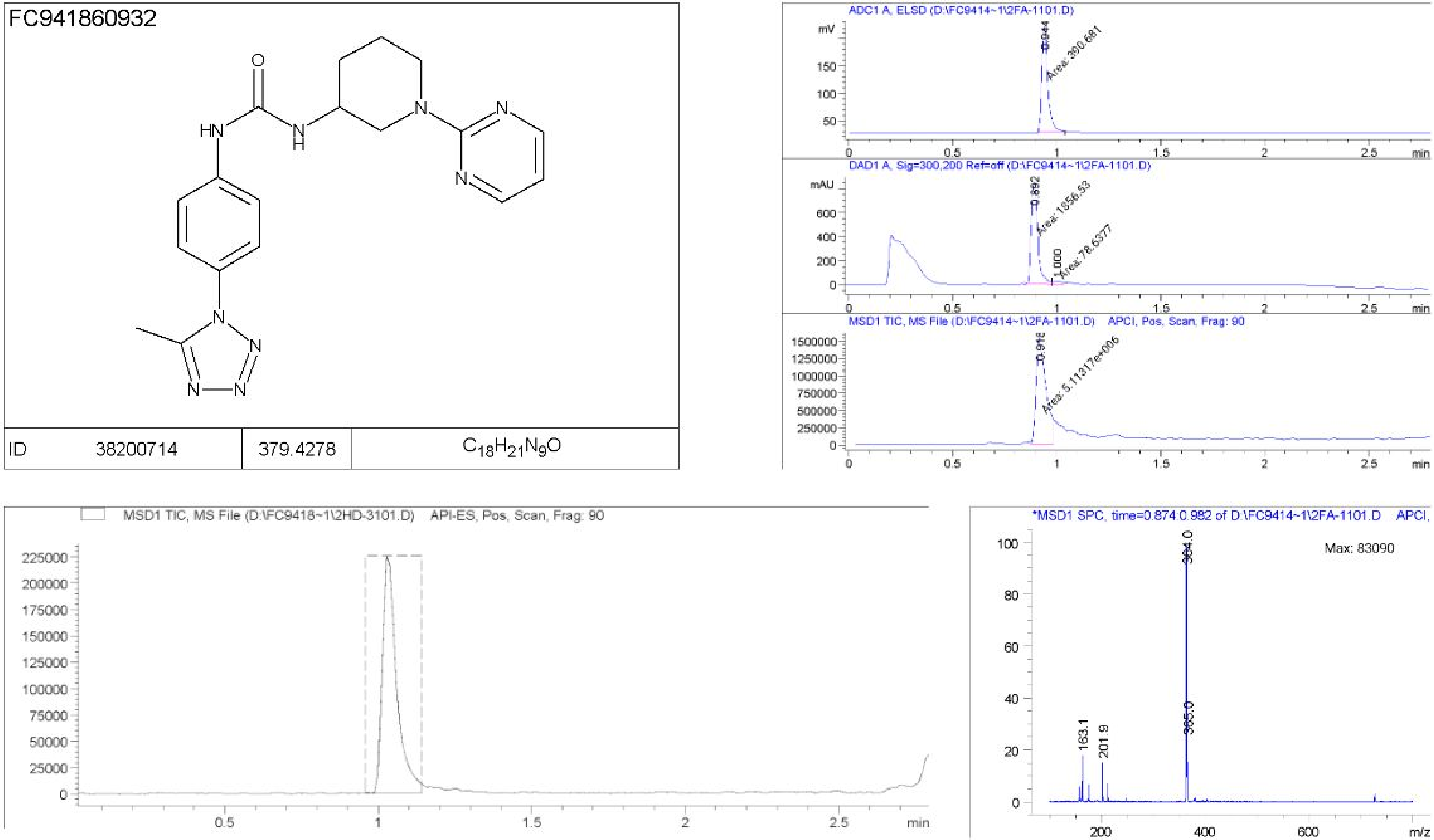

Figure S8. LC-24 (N-[4-(5-methyl-1H-tetrazol-1-yl)phenyl]-N'-(1-pyrimidin-2-ylpiperidin-3-yl)urea)

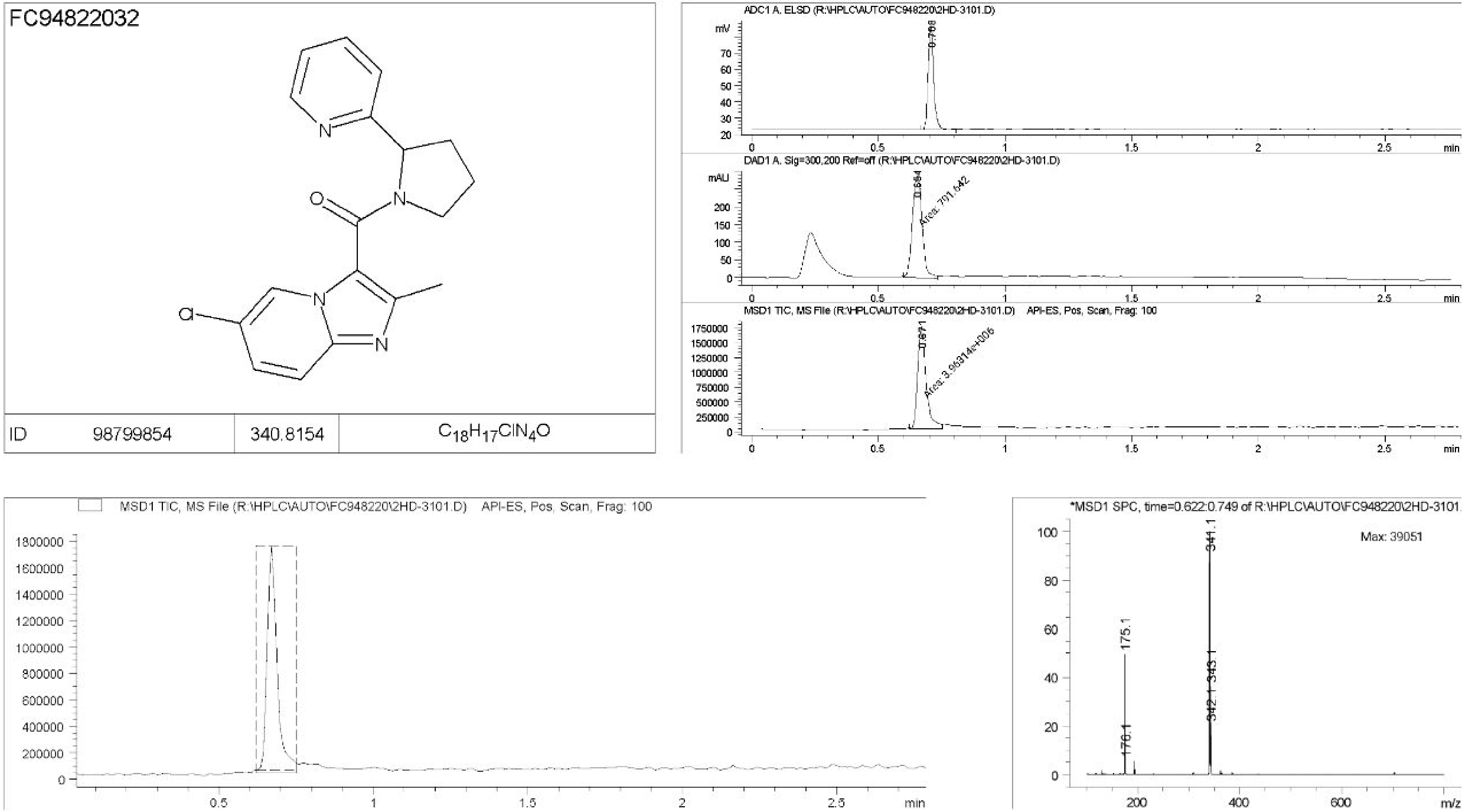

Figure S9. LC-25 (6-Chloro-2-Methyl-3-{[2-(2-Pyridinyl)-1-Pyrrolidinyl]Carbonyl}Imidazo[1,2-A]Pyridine).

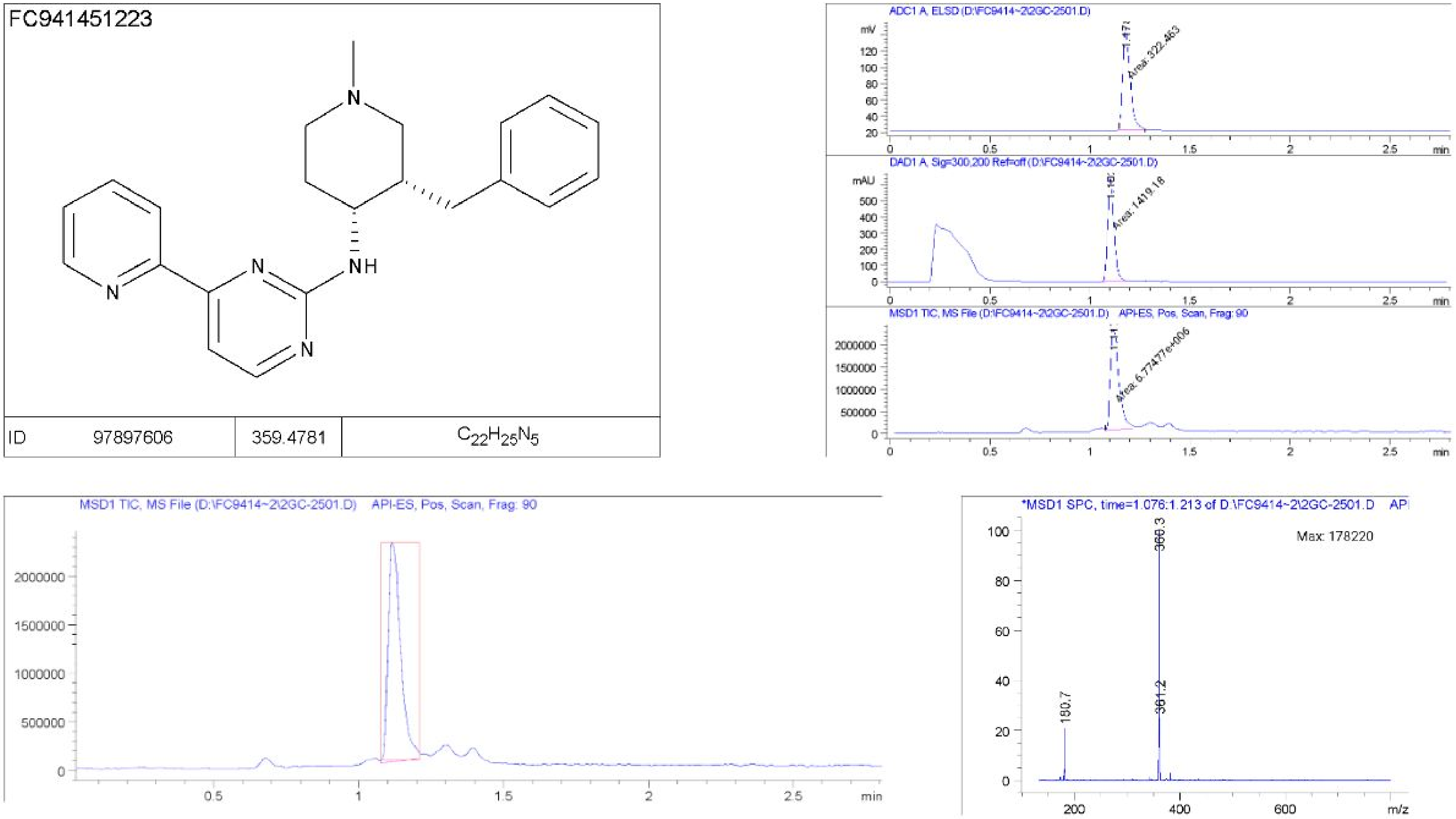

Figure S10. LC-26 (2-methyl-6-{1-[4-(2-pyridinyl)-2-pyrimidinyl]-4-piperidinyl}-4-pyrimidinol).

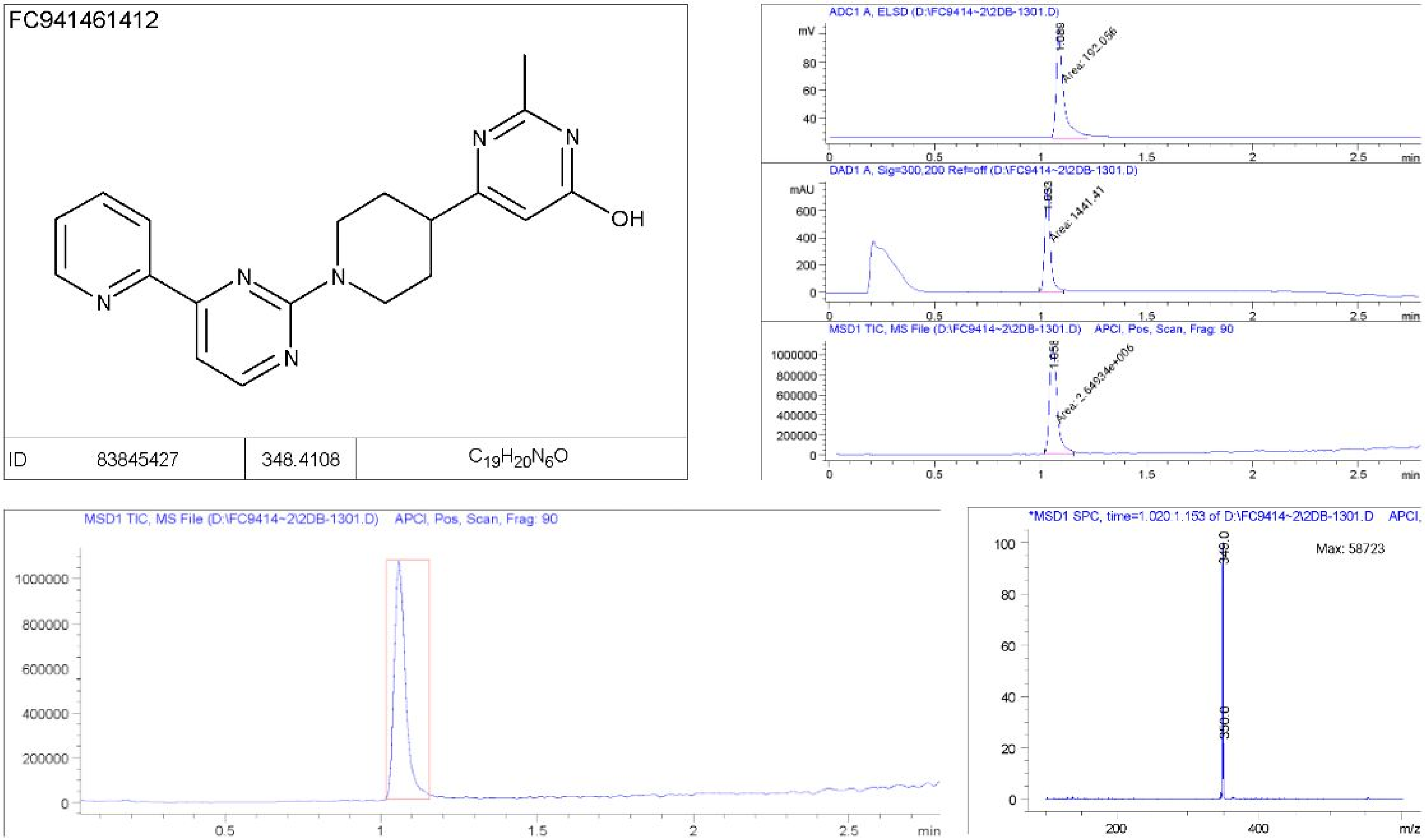

Figure S11. LC-27 (2-methyl-6-{1-[4-(2-pyridinyl)-2-pyrimidinyl]-4-piperidinyl}-4-pyrimidinol).

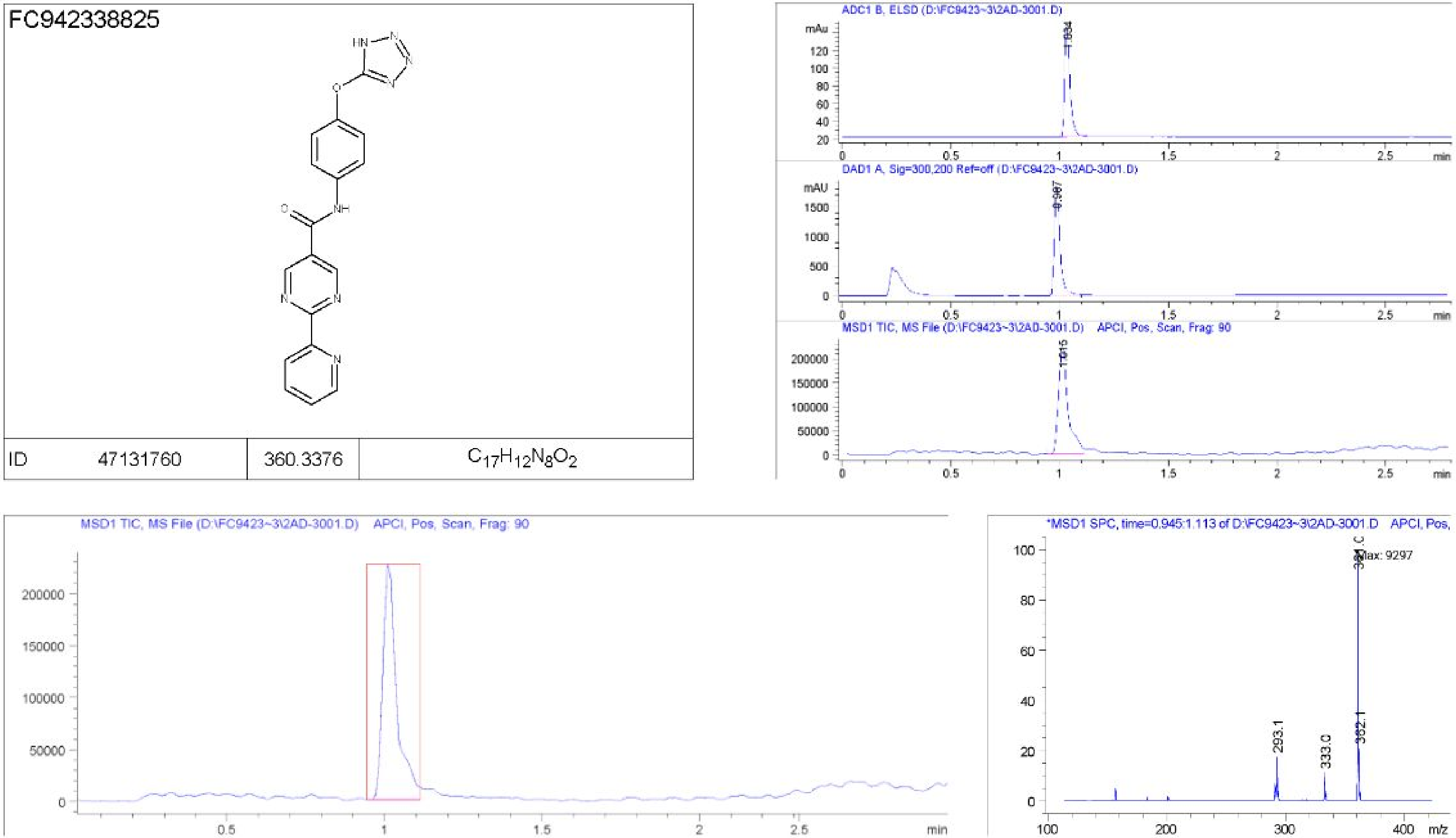

Figure S12. LC-28 (2-pyridin-2-yl-N-[4-(1H-tetrazol-5-yloxy)phenyl]pyrimidine-5-carboxamide).

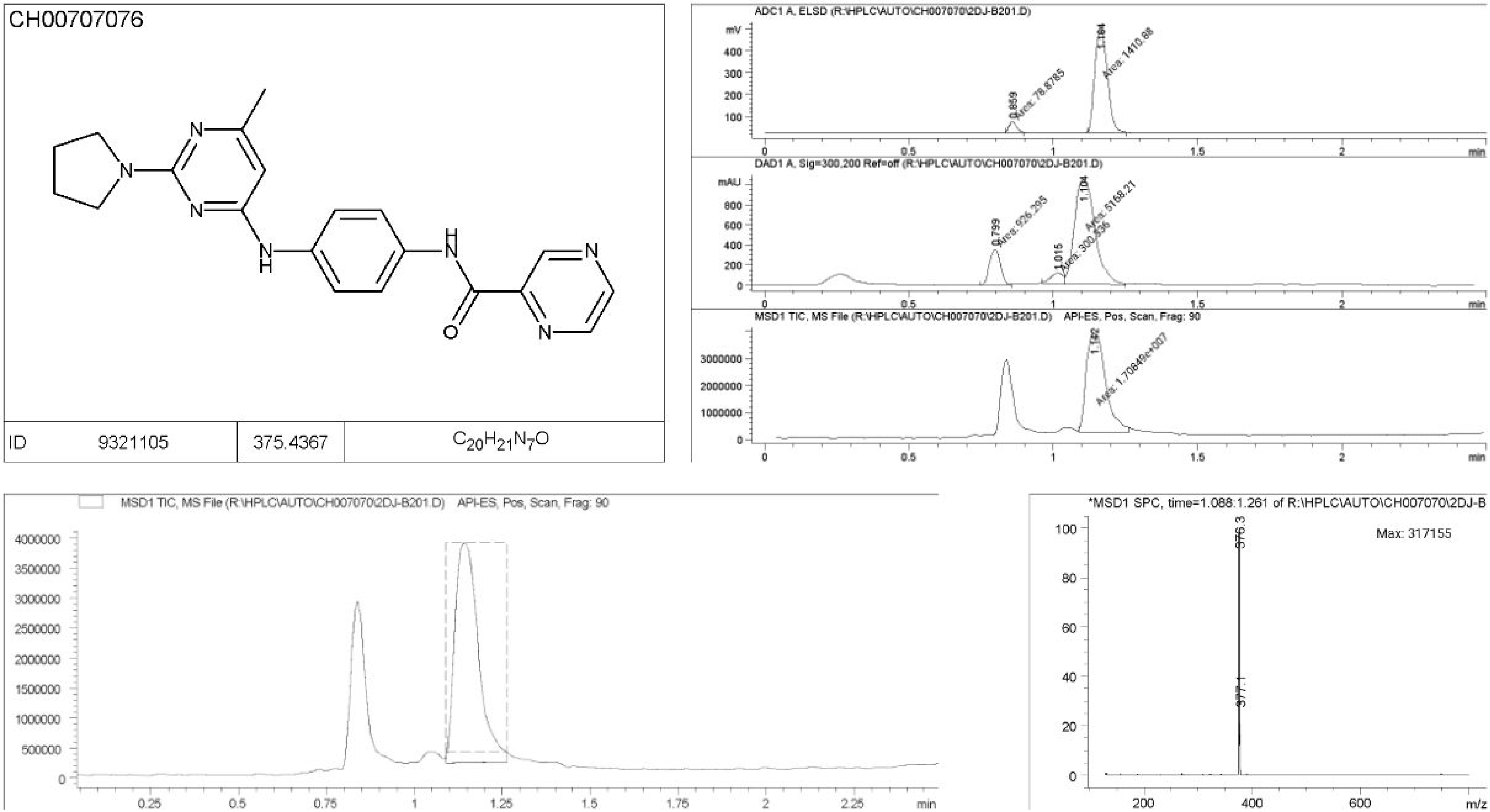

Figure S13. LC-29 (N-(4-{[6-methyl-2-(1-pyrrolidinyl)-4-pyrimidinyl]amino}phenyl)-2-pyrazinecarboxamide).

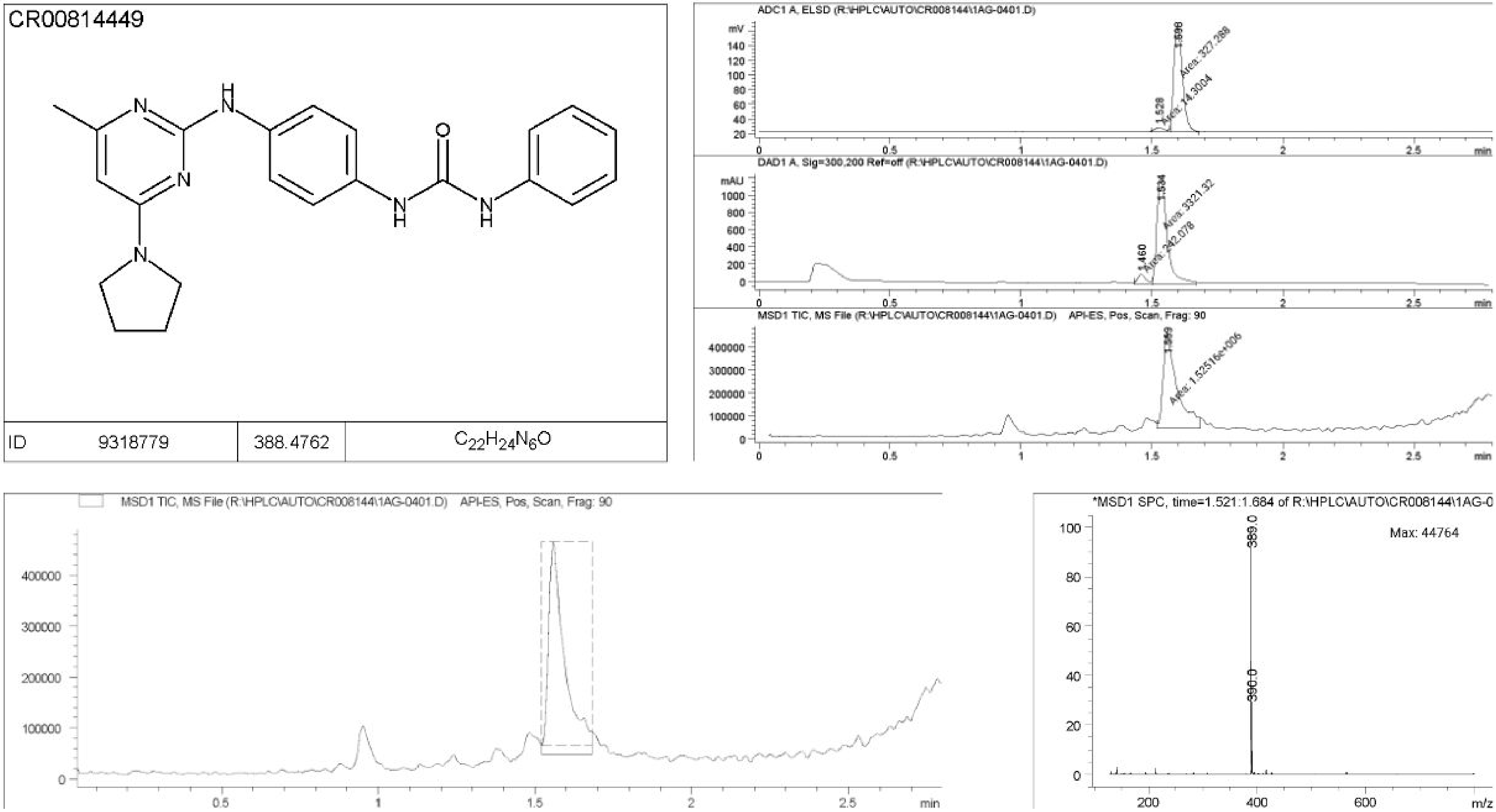

Figure S14. LC-30 (N-(4-{[4-methyl-6-(1-pyrrolidinyl)-2-pyrimidinyl]amino}phenyl)-N'-phenylurea).

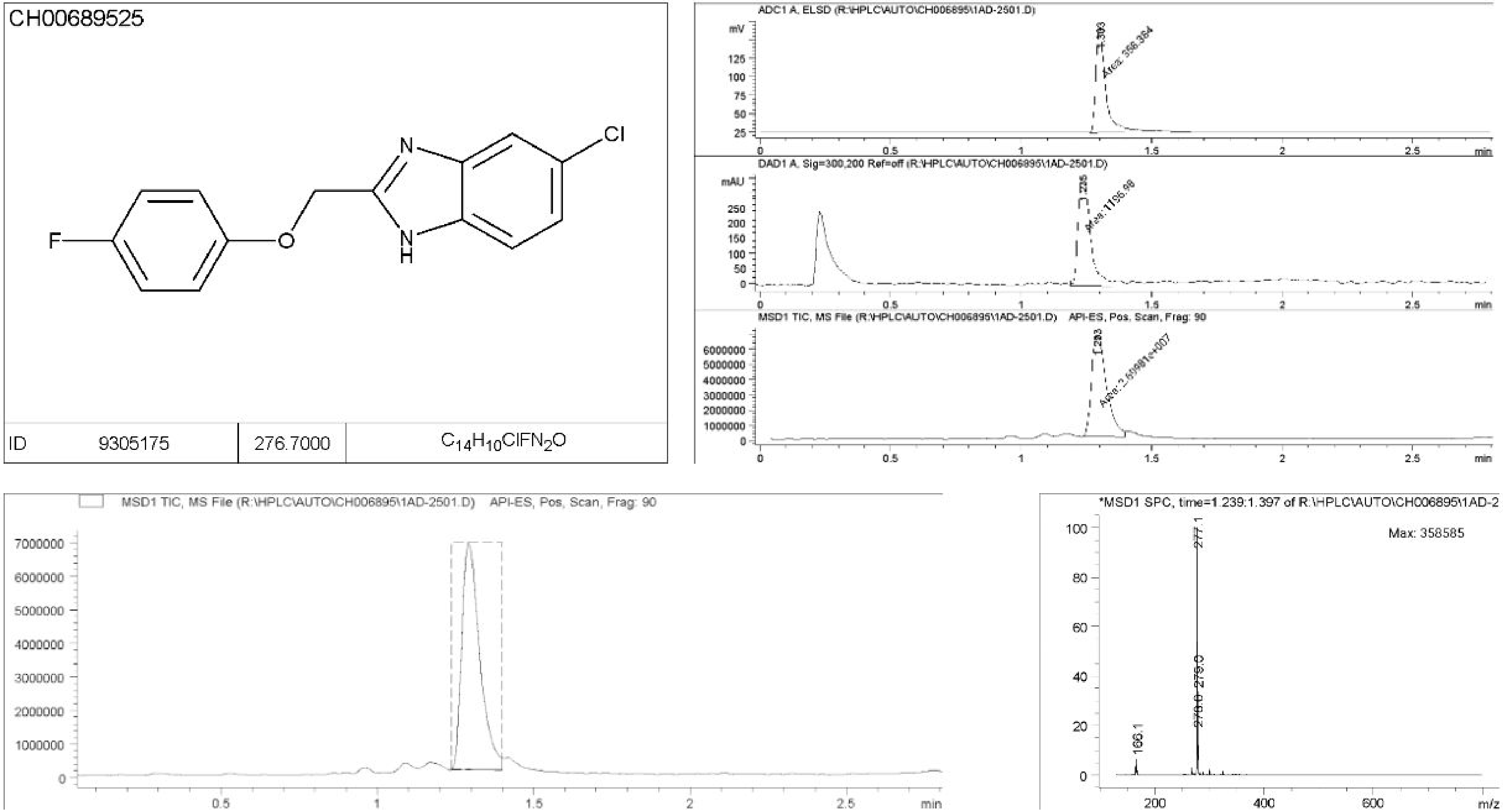

Figure S15. LC-31 (5-chloro-2-[(4-fluorophenoxy)methyl]-1H-benzimidazole).

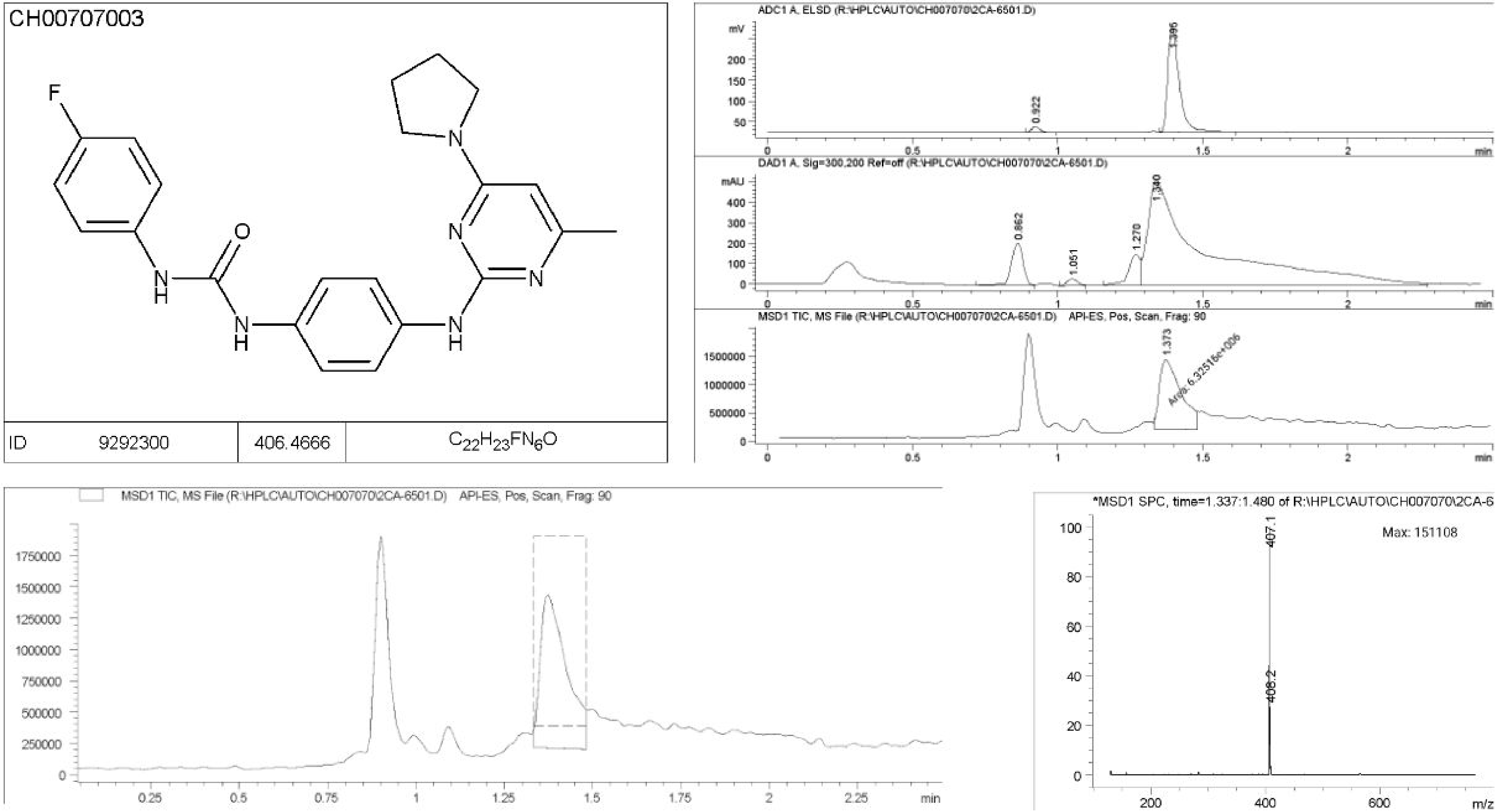

Figure S16. LC-32 (N-(4-fluorophenyl)-N'-(4-{[4-methyl-6-(1-pyrrolidinyl)-2-pyrimidinyl]amino}phenyl)urea).

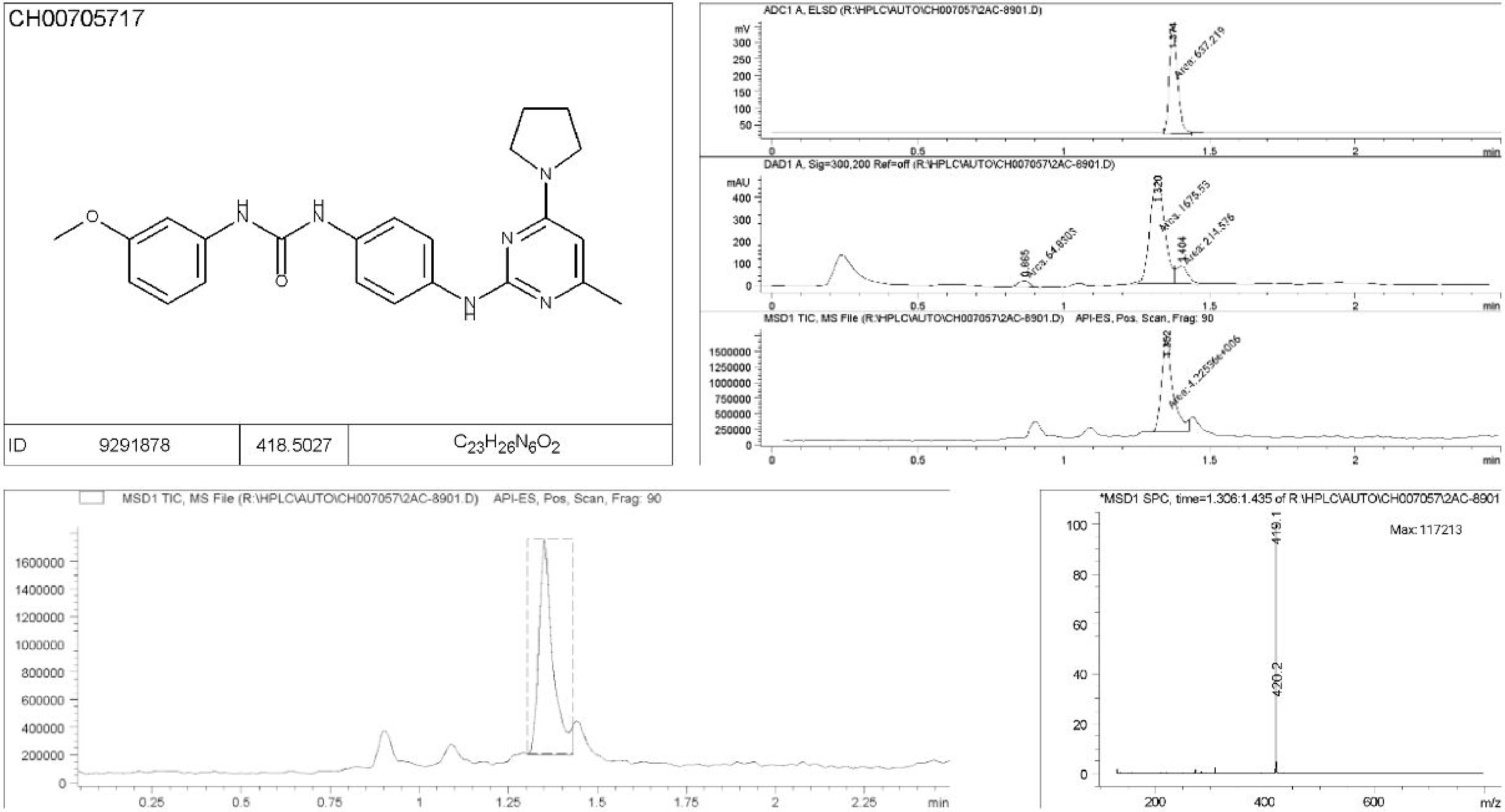

Figure S17. LC-33 (N-(3-methoxyphenyl)-N'-(4-{[4-methyl-6-(1-pyrrolidinyl)-2-pyrimidinyl]amino}phenyl)urea).

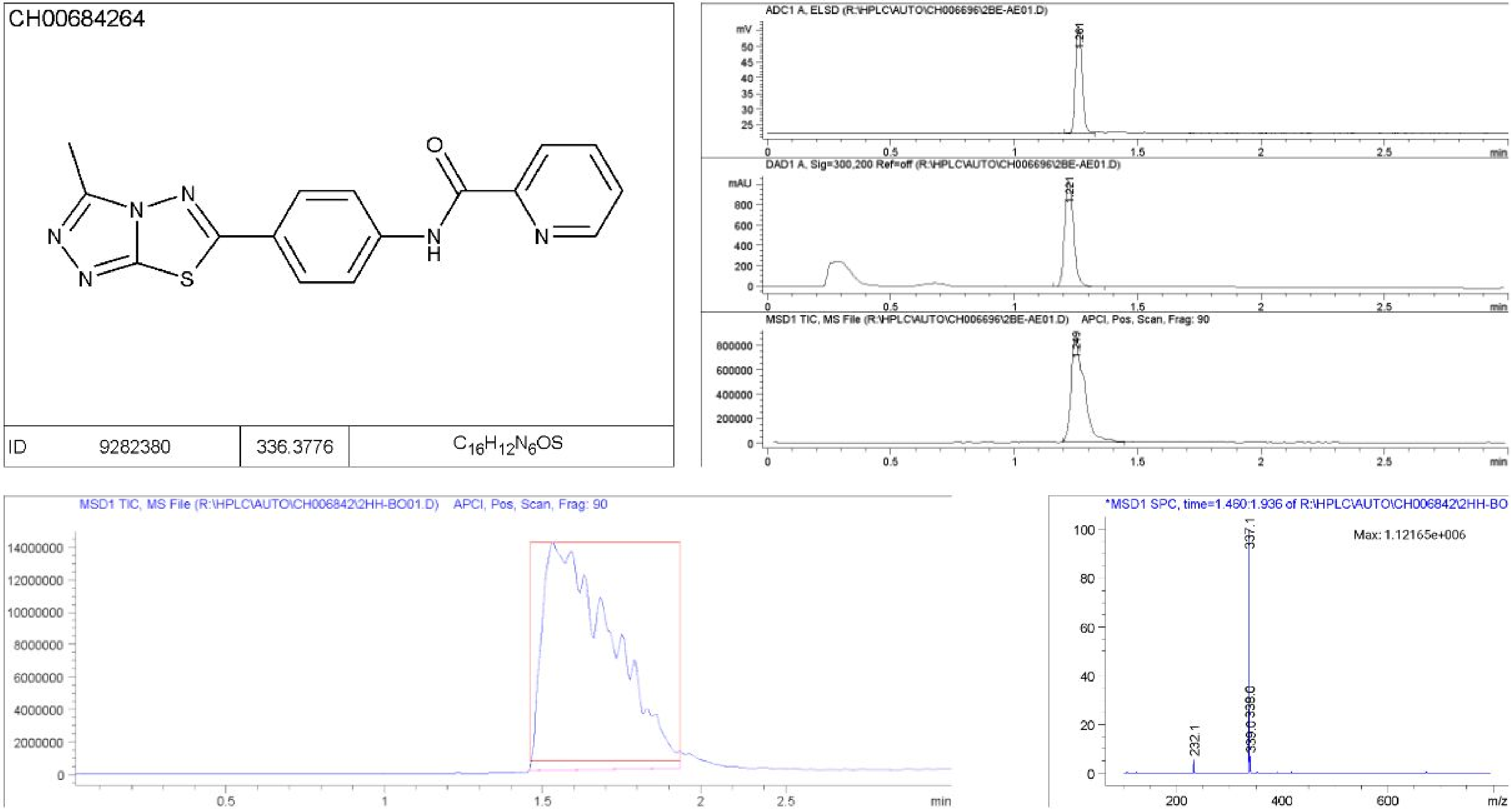

Figure S18. LC-34 (N-[4-(3-methyl[1,2,4]triazolo[3,4-b][1,3,4]thiadiazol-6-yl)phenyl]-2-pyridinecarboxamide)

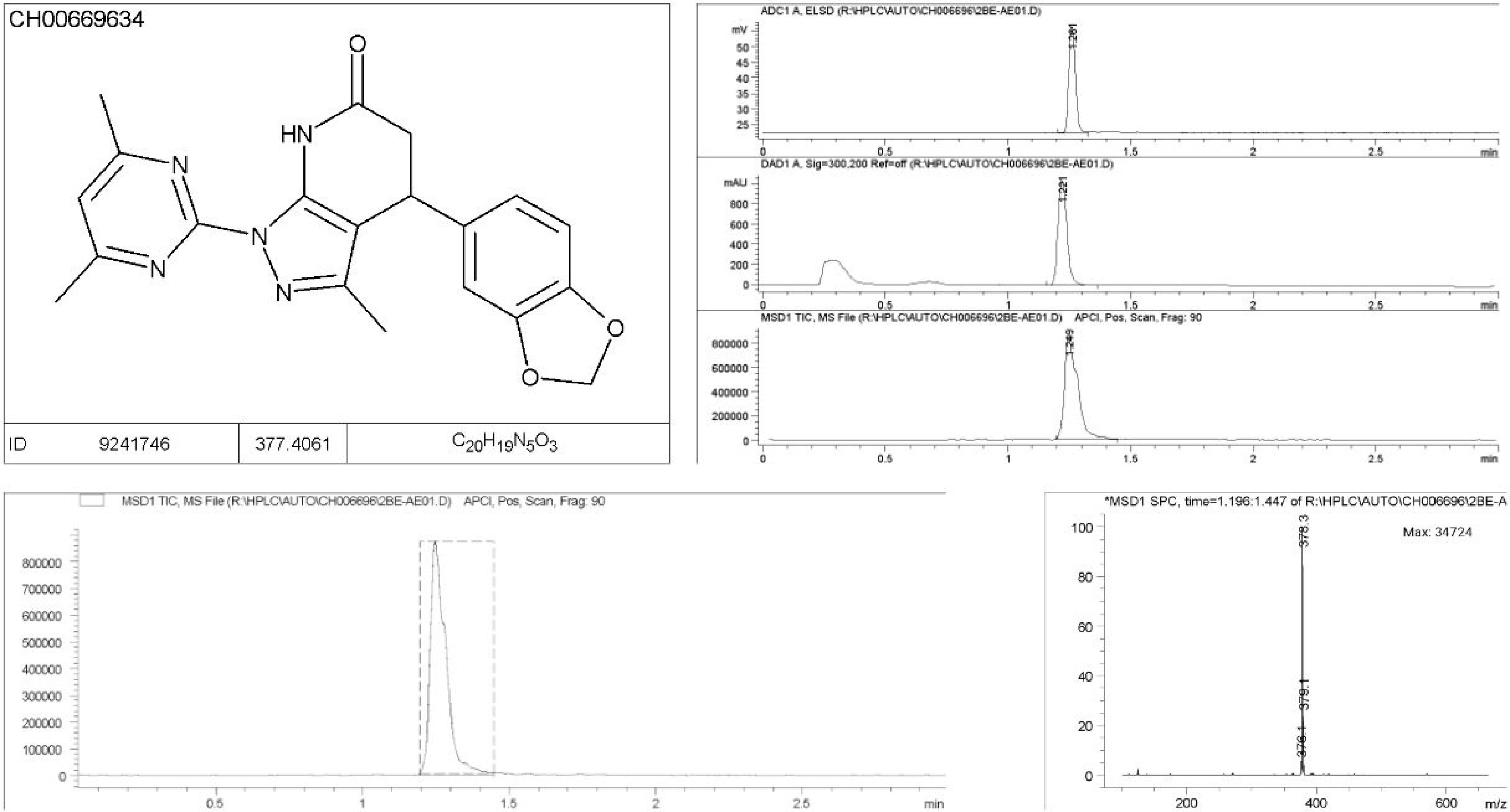

Figure S19. LC-35 (4-(1,3-benzodioxol-5-yl)-1-(4,6-dimethyl-2-pyrimidinyl)-3-methyl-1,4,5,7-tetrahydro-6H-pyrazolo[3,4-b]pyridin-6-one).

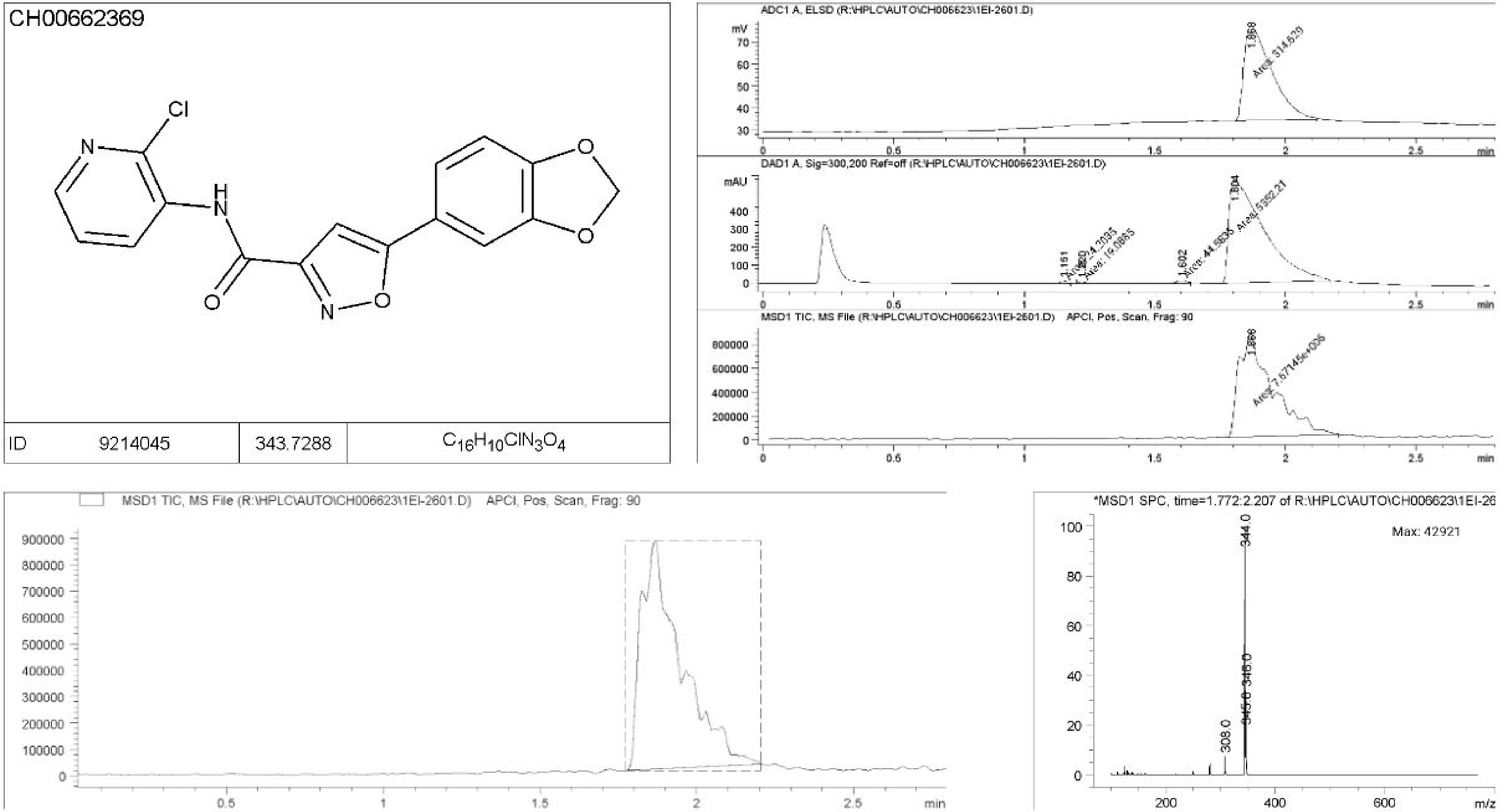

Figure S20. LC-36 (5-(1,3-benzodioxol-5-yl)-N-(2-chloro-3-pyridinyl)-3-isoxazolecarboxamide).

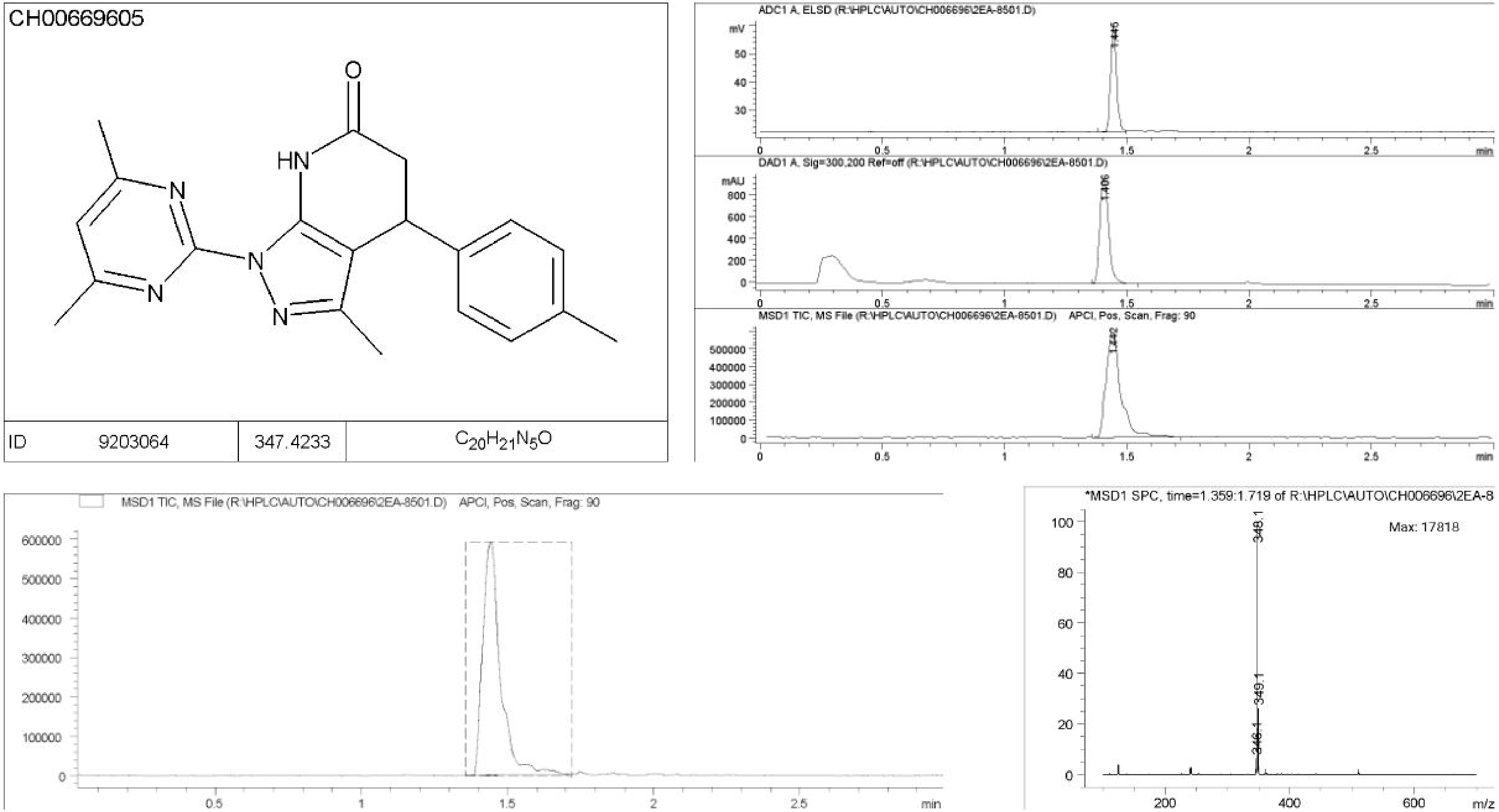

Figure S21. LC-37 (1-(4,6-dimethyl-2-pyrimidinyl)-3-methyl-4-(4-methylphenyl)-1,4,5,7-tetrahydro-6H-pyrazolo[3,4-b]pyridin-6-one).

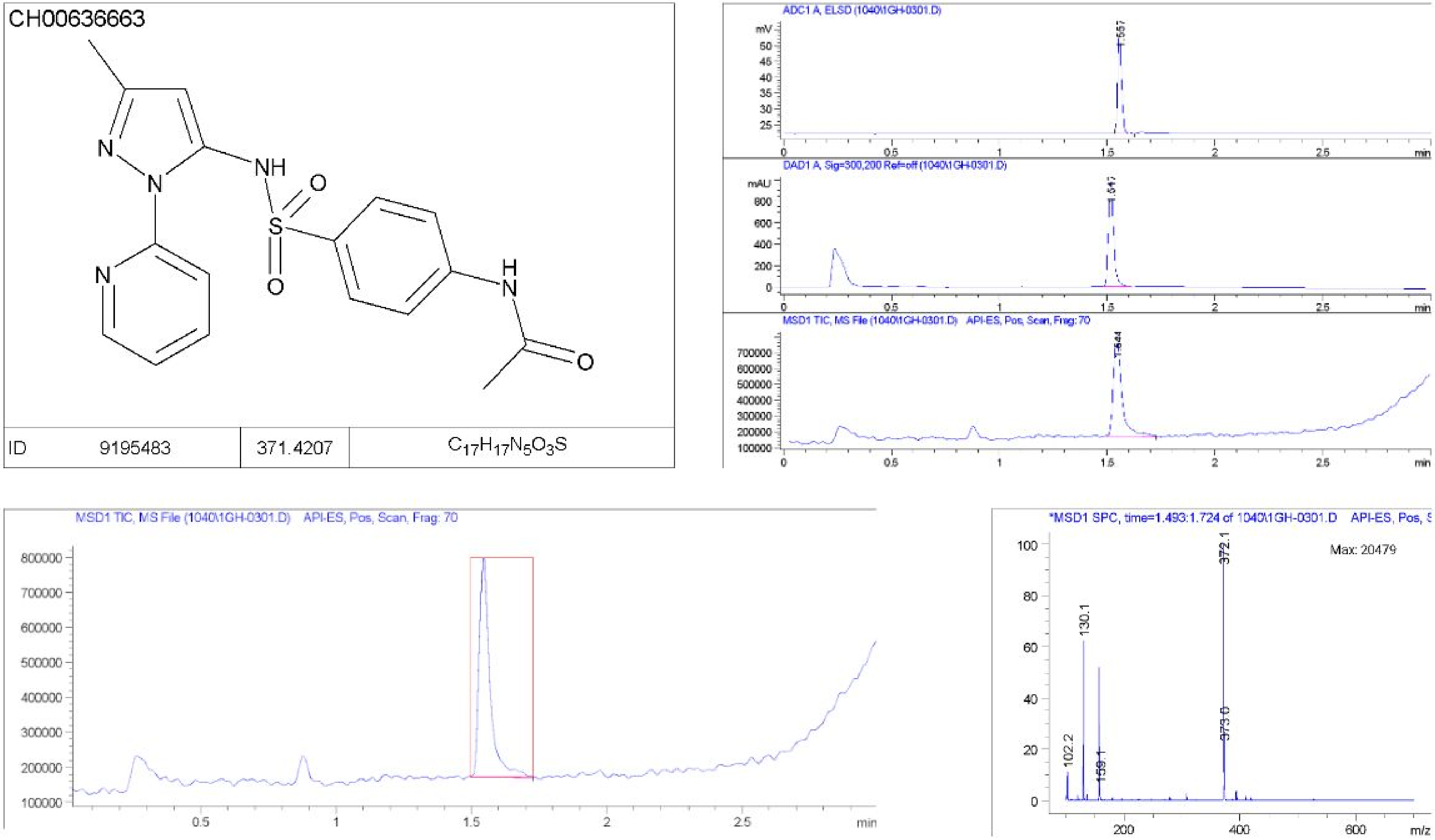

Figure S22. LC-38 (N-[4-({[3-methyl-1-(2-pyridinyl)-1H-pyrazol-5-yl]amino}sulfonyl)phenyl]acetamide).
